# Characterization of oxidative stress-induced *cgahp*, a gene coding for alkyl hydroperoxide reductase, from industrial importance *Corynebacterium glutamicum*

**DOI:** 10.1007/s10529-023-03421-8

**Published:** 2023-08-22

**Authors:** Meiru Si, Mengdie Hu, Mingfei Yang, Zhaoxin Peng, Donghan Li, Yuying Zhao

**Affiliations:** 1 College of Life Sciences, Qufu Normal University, Qufu, 273165 Shandong China; 2 College of Life Science and Agronomy, Zhoukou Normal University, Zhoukou, 466001 Henan China

**Keywords:** Alkyl hydroperoxide reductase, Peroxide stress, OasR, Corynebacterium glutamicum

## Abstract

Alkyl hydroperoxide reductase (Ahp), comprised of four different subunits AhpC, AhpD, AhpE, and AhpF, is a thiol-based antioxidative enzyme with the ability to protect bacteria against oxidative stress. Functionally, AhpC and AhpE considered as peroxidases directly detoxify peroxides, while AhpD and AhpF as oxidoreductases restore oxidized peroxidases to their reduced form. *Corynebacterium glutamicum ncgl0877* encodes a putative Ahp with a unique Cys-Pro-Phe-Cys (C-P-G-C) active-site motif, similar with those of the thiol-disulfide oxidoreductases such as thioredoxin (Trx), mycoredoxin-1 (Mrx1) and AhpD. However, its physiological and biochemical functions remain unknown in *C. glutamicum*. Here, we report that NCgl0877, designated CgAhp, is involved in the protection against organic peroxide (OP) stress. The *cgahp*-deleted strain is notably more sensitive to OP stress. The *cgahp* expression is controlled by a MarR-type transcriptional repressor OasR (organic peroxide- and antibiotic-sensing regulator). The physiological role of CgAhp in resistance to OP stresses is corroborated by its induced expression under stresses. Although CgAhp has a weak peroxidase activity toward OP, it mainly supports the OP-scavenging activity of the thiol-dependent peroxidase preferentially linked to the dihydrolipoamide dehydrogenase (Lpd)/dihydrolipoamide succinyltransferase (SucB)/NADH system. The C-P-G-C motif of CgAhp is essential to maintain the reductase activity. In conclusion, our study identifies CgAhp, behaving like AhpD, as a key disulfide oxidoreductase involved in the oxidative stress tolerance and the functional electron donor for peroxidase.

**Supplementary Information:**

The online version contains supplementary material available at 10.1007/s10529-023-03421-8.

## Introduction

Reactive oxygen species (ROS) are not only toxic byproducts of oxygen from normal metabolic process, such as photosynthesis and respiration metabolism, but also induced by unfavorable adverse environmental stimuli, such as oxidants, low pH, heavy metal, high temperature, diamide and antibiotic (Dalle-Donne et al. 2008). ROS, such as hydrogen peroxide (H_2_O_2_), superoxide anion (O_2_^•−^), and organic peroxides, are highly reactive molecules that are capable of reacting with protein, DNA and cell membranes, modifying them and causing different degrees of damaging, and even lead to the disruption of intracellular redox homeostasis and provoke oxidative stress (Den Hengst and Buttner 2008). ROS modification of the free thiol of the cysteine molecule results in increasingly oxidized forms, from disulfide bond to sulfenic (–SOH), sulfinic (–SO_2_H), sulfonic (–SO_3_H) acid, mixed disulfides between cysteine thiol of a protein and a different molecule, such as the tripeptide glutathione, L-g-glutamyl-L-cysteinyl-glycine (GSH in its reduced form) (Ying et al. 2007). Under certain conditions formation of a disulfide bond between two cysteine thiols can inactivate the protein due to alteration of the active protein structure or because specific oxidation of the thiol(s) is required for protein activity. To counteract ROS toxicity, cells produced various antioxidant enzymes, including directly ROS-scavenging terminal peroxidases, oxidized proteins-repairing oxidoreductases and regulatory proteins, for continuously monitoring the change in the intracellular redox state and facilitating the proper folding of proteins (Liochev 2013).

The enzymatic scavenging system for ROS involves a number of enzyme-catalyzed reactions in different cellular compartments. Thiol-based peroxidases, which have been found from archaea, lower prokaryotes to higher eukaryotes, constitute a large family including peroxiredoxin (Prx), glutathione peroxidase (GPx), and organic hydroperoxide resistance (Ohr) (Chao et al. 2009). Thiol-based peroxidases metabolize peroxides via conserved active cysteine residues, which undergo oxidation. To complete the catalytic cycle, the Cys residues must be reduced. Various peroxidases rely on diverse reducing systems, including alkyl hydroperoxide reductase subunit F (AhpF); thioredoxin (Trx) and thioredoxin reductase (TrxR); tryparedoxin, trypanothione, and trypanothione reductase; mycoredoxin-1 (Mrx1), mycothione reductase (Mtr), mycothiol (MSH); alkyl hydroperoxide reductase subunit D (AhpD), dihydrolipoamide dehydrogenase (Lpd), dihydrolipoamide succinyltransferase (SucB); ribonucleotide reductases H (NrdH) and thioredoxin reductase (TrxR); thiol disulfide interchange protein (DsbA)-like Mrx1, Mtr, MSH; cyclophilin (Lu and Holmgren 2014; Lee et al. 2001; Poole 2005; Bryk et al. 2002; Li et al. 2021; Rosado et al. 2017). Oxidized peroxidase disulfide oxidoreductases, such as AhpF, Trx, Mrx1, DsbA-like Mrx1, and AhpD, are the core member of the reducing systems, directly interact with oxidized peroxidases and transfer the electron to oxidized peroxidases.

Ahp, comprised of four different subunits alkyl hydroperoxide reductase subunit C (AhpC), AhpD, alkyl hydroperoxide reductase subunit E (AhpE), and AhpF, is the known member of a class of disulfide oxidoreductases (Tartaglia et al. 1990) and the thiol-dependent Prx family with the abilities to propagate free-radical chain reactions and to directly detoxify ROS (Wood et al. 2004). AhpC and AhpE in bacteria catalyze the reduction of H_2_O_2_, tertbutyl hydroperoxide (*t*-BHP), cumene hydroperoxide (CHP) and peroxynitrite (Bryk et al. 2000; Zhang et al. 2019; Hugo et al. 2014). AhpF is a flavoprotein with oxidoreductase activity that restores oxidized AhpC to its reduced form (Poole 2005). In some *ahpF*-absent bacteria, *ahpD* has no sequence similarity with either *ahpC* or *ahpF* but serves an analogous role to *ahpF* in *mycobacteria* and *Streptomyces avermitilis* (Liu et al. 2016). Therefore, *ahp* expression plays an important role in peroxide resistance (oxidative stress). Bioinformatics analysis revealed that non-pathogenic *C. glutamicum*, which is often used as a model to study and a widespread Gram-positive bacterium of industrial importance, contains three Ahp homologs (NCgl2286 for AhpD1, NCgl2349 for AhpD2, and NCgl0877 for the putative Ahp) (Portevin et al. 2005). Although *C. glutamicum* AhpD1 and AhpD2 contributed to regenerate a variety of thiol-dependent peroxidase in the decomposition of peroxide by linking the Lpd/SucB/NADH system through the cyclization of their own active site dithiol to the oxidized disulphide (Su et al. 2019), scant information on NCgl0877 has been reported. Recently, Si et al. (2020) found NCgl0877 was one of the main targets of OasR (organic peroxide- and antibiotic-sensing regulator), which is strongly linked to the oxidative stress response in *C. glutamicum*. It has a Cys-Pro-Gly-Cys (C-P-G-C) active-site motif embedded within its thioredoxin fold domain, which was different from the C-G-T-C or C-V-Y-C motif in AhpDs. Previous studies showed that the difference of two intervening residues between two cysteines in the catalytic CXXC motif makes oxidized peroxidase disulfide reductases have different enzymatic and substrate preference properties (Rosado et al. 2017). Therefore, the discovery of new C-P-G-C active site motif prompts us to investigate what NCgl0877 of *C. glutamicum*, designated CgAhp, behaves like, its contributions in *C. glutamicum* to ROS resistance, and the gene expression pattern.

## Materials and methods

### Bacterial strains and growth conditions

The bacterial strains and plasmids used in this study were listed in Supplementary Table S1. Luria–Bertani (LB) broth and LB agar plates were used for growing *Escherichia coli* or *C. glutamicum* RES167. *E. coli* and *C. glutamicum* were cultivated at 37 °C and 30 °C under vigorous agitation (220 rpm) as previously reported, respectively (Shen et al. 2005). Brain–heart broth medium containing 0.5 M sorbitol (BHIS) was used for producing and maintaining mutant of a gene in *C. glutamicum* RES167 strain (Shen et al. 2005). For creating *a cgahp* gene (*ncgl0877*) in-frame deletion in *C. glutamicum* RES167 strain, the pK18*mobsacB-*Δ*cgahp* plasmids were introduced into *C. glutamicum* RES167 strain through electroporation and then integrated into the chromosome of *C. glutamicum* RES167 strain through homologous recombination (Shen et al. 2005). Complementation in Δ*cgahp* mutants was carried out using the pXMJ19-*cgahp* derivatives, which were transformed into Δ*cgahp* mutants by electroporation (Su et al. 2019). 0.5 mM isopropyl β-D-thiogalactopyranoside (IPTG) (Sigma-Aldrich) was added into medium to induce the expression of *cgahp* gene on the pXMJ19-*cgahp* derivatives in complementary strains. *E. coli* DsbA was from Xinbosheng Biological Technology Co., Ltd. (Shenzhen, Guangdong, China). Antibiotics were added at the following concentrations: kanamycin (KAN), 50 µg ml^−1^ for *E. coli* and 25 µg ml^−1^ for *C. glutamicum*; nalidixic acid (NAL), 40 µg ml^−1^ for *C. glutamicum*; chloramphenicol (CHL), 20 µg ml^−1^ for *E. coli* and 10 µg ml^−1^ for *C. glutamicum*.

### Plasmid construction

Primers used in this study were listed in Supplementary Table S2. The *cgahp* gene (*ncgl0877*) region of *C. glutamicum* was amplified by PCR with primer pair Ocgahp-F and Ocgahp-R from genomic DNA of *C. glutamicum* RES167 and cloned into pET28a vector between *Eco*RI and *Xho*I sites, yielding pET28a-*cgahp*.

The suicide plasmid pK18*mobsacB*-Δ*cgahp* was obtained by introducing a 483-bp in-frame deletion by two-step recombination as described previously (Su et al. 2018). First, two DNA fragments flanking and overlapping the distal parts of *cgahp* were amplified from genomic DNA of *C. glutamicum* RES167. Two oligonucleotide primer pairs namely Dcgahp-F1/Dcgahp-R1 and Dcgahp-F2/Dcgahp*-*R2 listed in Supplementary Table S2 were made and used for the amplification (Sangon Biotech Co., Ltd., Shanghai, China). Primer pair Dcgahp-F1/Dcgahp-R1 was used to amplify the *cgahp*’s upstream 746-bp fragment; while primer pair Dcgahp-F2/Dcgahp*-*R2 was used to amplify the *cgahp′*s upstream 720-bp fragment. The upstream and downstream fragments were ligated together using overlap PCR with the primer pair Dcgahp-F1/Dcgdahp*-*R2. The resulting PCR products were cut with *Eco*RI and *Bam*HI and subsequently cloned into pK18*mobsacB* vector between *Eco*RI and *Bgl*II sites to produce plasmid pK18*mobsacB*-Δ*cgahp*.

For obtaining pXMJ19-*cgahp*, primer pair Ccgahp-F/Ccgahp-R was used to amplify DNA fragments of open reading frames region of *cgahp* gene from *C. glutamicum* genomic DNA. The resulting DNA fragments were cut with *Sal*I and *Bam*HI and then cloned into pXMJ19 vector between *Sal*I and *Bam*HI sites.

To create the *cgahp*:*C42S* mutation construct, site-directed mutagenesis was carried out by two rounds of PCR (Su et al. 2018). Briefly, two oligonucleotide primer pairs namely Dcgahp-F1/cgahp-C42S-R and cgahp-C42S-F/Dcgahp*-*R2 listed in Supplementary Table S2 were designed and synthesized. In the first round of PCR, primer pair Dcgahp-F1/cgahp-C42S-R was used to amplify the 5′ primer region of *cgahp* (Fragment I); while primer pair cgahp-C42S-F/Dcgahp-R2 was used to amplify the 3′ prime region of *cgahp* (Fragment II). The second round of PCR was performed by using Ocgahp-F/Ocgahp-R as primer pair and fragment I and fragment II as templates to get the *cgahp*:*C42S* fragment. The *Bam*HI and *Sal*I cut *cgahp*:*C42S* DNA fragments was cloned in pET28a plasmid digested with similar enzymes to create plasmid pET28a-*cgahp*:*C42S*. The *cgahp*:*C42S* fragments were obtained using a similar procedure as described above and cloned into pET28a to produce pET28a-*cgahp:C45S* derivatives.

For obtaining the *lacZY* fusion reporter vector pK18*mobsacB-P*_*cgahp*_::*lacZY*, the fusion of *cgahp* promoter to the *lacZY* reporter gene by overlap PCR was perform. First, two oligonucleotide primer pairs namely *P*_*cgahp*_-F/*P*_*cgahp*_-R and *lacZY*-F/*lacZY*-R were designed in the first round of PCR to amplify the 752-bp *cgahp* promoter DNA fragments (corresponding to nucleotides + 12 to − 740 relative to the translational start codon (ATG) of *cgahp* gene) and the *lacZY* DNA fragments, respectively. Second, *P*_*cgahp*_-F/*lacZY*-R as primers and the first round PCR products as templates were used to carry out the second round of PCR, and the resulting fragments cut with *Sma*I and *Pst*I were inserted into pK18*mobsacB* between *Sma*I and *Pst*I sites to get the pK18*mobsacB-P*_*cgahp*_::*lacZY* fusion construct (Su et al. 2018).

The fidelity of all constructs was confirmed by DNA sequencing (Sangon Biotech, Shanghai, China).

### Overexpression and purification of recombinant protein

*E. coli* BL21(DE3) cells harboring the pET28a derivatives plasmid were grown in LB medium containing kanamycin (50 µg ml^−1^) at 37 °C with shaking at 220 rpm. After cells were grown to an OD_600_ nm of 0.6, 0.5 mM IPTG was added and then the cultures were cultivated for an additional 10 h at 22 °C. Cells were harvested by centrifugation at 4 °C. Cell pellets was suspended in 30 ml lysis buffer [10 mM Tris (pH 6.8), 10% glycerol and 10 mM β-mercaptoethanol (β-ME)], sonicated, and centrifuged at 10,000×*g* for 60 min. Target proteins in the supernatant were purified with the His•Bind Ni-NTA resin (Novagen, Madison, WI) according to manufacturer’s instructions. The purified His_6_-tag proteins were dialyzed against PBS at 4 °C and concentrated for further experiments [> 95% purity as estimated by sodium dodecyl sulfate-polyacrylamide gel electrophoresis (SDS-PAGE)].

### Agar-based disk diffusion assay

Disk diffusion assays were performed for oxidants, antibiotics, alkylating agents, and heavy metal according to Li et al. (2021). Briefly, bacterial strains were grown to the mid-log phase and 100 µl of culture containing about 10^7^ cfu was spread onto 20-ml LB agar plates. Paper disks soaked with 10 µl of a stock solution of reagents were placed on top of the agar. Stock solutions were 200 mM H_2_O_2_, 0.5 mM hypochlorous acid (HClO), 5 mM diamide, 11 mM cumene hydroperoxide (CHP), 60 mM tert-butyl hydroperoxide (*t*-BHP), 70 mM 1-chloro-2,4-dinitrobenzene (CDNB), 1 mM iodoacetamide (IAM), 25 mg ml^−1^ streptomycin (STR), 5 mg ml^−1^ ciprofloxacin (CIP), 0.5 mM cadmium chloride (CdCl_2_), and 10 mM nickel sulfate (NiSO_4_). The disks were allowed to dry and the plates were incubated for 2 to 3 days at 30 °C. The diameter of the inhibition zone was measured. Experiments were performed with at least three independent biological replicates.

### NBD-Cl analysis of the sulfenic acid state

To study the formation of cysteine sulfenic acid (Cys-SOH) as a reaction intermediate, CgAhp:C42S and CgAhp:C45S labeled with 4-chloro-7-nitrobenzofurazan (NBD-Cl) were assayed as described previously (Ellis and Poole 1997) with minor modifications. CgAhp:C42S and CgAhp:C45S were pretreated with 50 mM DTT for 30 min and then were made anaerobic by repeated flushing with argon gas and vacuum in alternating cycles for 20 min. An anaerobic solution of NBD-Cl [25 mM in dimethyl sulfoxide (DMSO)] was prepared by bubbling argon through the solution for 10 min. Under anaerobic conditions, CgAhp:C42S and CgAhp:C45S were divided into equally three portions (final concentration of 20 µM), the first of which was treated with 20 µM CHP, the second of which was treated with 20 µM CHP and 500 µM MSH, while the last of which was directly used as an untreated sample (negative control). The CHP-treated and untreated proteins were incubated with NBD-Cl (5 mM) for 30 min at 25 °C in the dark. Excess NBD-Cl was removed by ultrafiltration, and the protein samples were analyzed at 200 to 600 nm (DU 7500 diode array spectrophotometer; Beckman, Fullerton, CA). Experiments were performed in triplicate.

### Quantitative analysis of sulfhydryl groups

Free sulfhydryl groups in CgAhp WT, CgAhp:C42S and CgAhp:C45S were measured using 5,5′-dithio-bis (2-nitrobenzoic acid) (DTNB) (Ellman 1959). After 20 µM proteins were treated with 20 µM CHP, 20 µM CHP + 500 µM MSH, and 50 mM DTT at room temperature for 30 min, respectively, followed by removing residual DTT or H_2_O_2_ with a PD10 desalting column (GE Healthcare, Piscataway, NJ). The resulting proteins (10 µM) were added to 2 mM DTNB in 50 mM Tris–HCl buffer (pH 8.0) and the absorbance at 412 nm was measured against a 2 mM DTNB solution as the reference. The amounts of reactive sulfhydryl groups were measured using the molar absorption coefficient of TNB at 412 nm (*Ɛ*_412_) of 13,600 M^−1^ cm^−1^ (Gething and Davidson 1972). Experiments were performed in triplicate.

### p*Ka* determination

The extinction coefficient of thiol groups (R-SH) at 240 nm was the main readout utilized to measure p*Ka* values of cysteine residues due to the lack of absorption of its un-ionized counterpart (R-S) in the same wavelength (Roos et al. 2007). To cover a broad pH range, a reaction mixture containing a poly-buffer solution composed of 10 mM sodium acetate, 10 mM sodium phosphate, 10 mM sodium borate, and 10 mM sodium citrate, pH 9.4, was used. For the oxidation of cysteine mutants, a 10-fold excess of CHP was used. Excess of CHP was removed by ultrafiltration. A final reaction mixture of 20 µM CgAhp (reduced or oxidized) was titrated with 100 mM HCl. The p*Ka* of CgAhp:C42S and CgAhp:C45S variants were determined in the same conditions as described for CgAhp WT. All of the measurements were performed in a Carry UV spectrophotometer (Agilent Technologies) precooled at 10 °C. The sigmoidal pH-dependent saturation curve was fitted to the Henderson-Hasselbalch equation (Roos et al. 2007), where *A*_exp_ was the experimental value *A*_240_/*A*_280_, *A*_SH_ was the *A*_240_/*A*_280_ value for the protonated form, and *A*_S_^−^ is the *A*_240_/*A*_280_ for the deprotonated form. The data were fitted to the following equation using GraphPad Prism version 5.

$$A_{{\exp }} = A_{{{\text{SH}}}} + \frac{{\left( {A_{{\text{S}}} - A_{{{\text{SH}}}} } \right)}}{{1 + 10^{{\left( {{\text{p}}K_{{\text{a}}} - {\text{pH}}} \right)}} }}$$

### Steady-state kinetics of oxidized CgAhp-S_2_ by TrxR/NADPH, MSH/Mtr/NADPH, and Lpd/SucB/NADH pathways

The assay mixture of TrxR/NADPH was prepared by diluting all components, excepts oxidized CgAhp in the Tris–HCl buffer pH 7.5 buffer to a final concentration of 3 µM TrxR and 500 µM NADPH. The MSH assay mixture consisted of 3 µM Mtr, 500 µM MSH and 500 µM NADPH. The Lpd assay mixture consisted of 3 µM Lpd, 3 µM SucB and 500 µM NADH. All concentrations were calculated taking into account the subsequent addition of oxidized CgAhp. The mixtures were incubated for 5 min at 37 °C in a 96-well plate. The assay was started by adding 50 µM of oxidized CgAhp in each well. The absorption at 340 nm was monitored. For each reaction mixture, a control well without oxidized CgAhp was added.

CgAhp-S_2_-dependent oxidation of NADPH or NADH in the TrxR/NADPH, MSH/Mtr/NADPH, or Lpd/SucB/NADH pathway was continuously monitored at 340 nm (*Ɛ*_340_ = of 6220 M^−1^ cm^−1^) in a 300 µl reaction mixture containing 50 mM Tris–HCl buffer (pH 7.5), 1 mM EDTA, varying concentrations of oxidized CgAhp-S_2_ (0–50 µM), and a reduced TrxR-generating system (3 µM TrxR and 500 µM NADPH), MSH system (3 µM Mtr, 500 µM MSH and 500 µM NADPH), or Lpd system (3 µM Lpd, 3 µM SucB and 500 µM NADH) as the possible electron donors. All reactions were carried out at 37 °C and started by the addition of oxidized CgAhp-S_2_ in a reaction mixture previously incubated for 5 min at 37 °C. Control measurements were performed in the absence of CgAhp-S_2_. Reactions were performed in duplicate. The *k*_cat_ and *K*_m_ values were obtained from a non-linear fit with the Michaelis–Menten equation using the program GraphPad Prism 5.

### Reductase activity of CgAhp toward MPx:C64S-S_2_ and prx:C84S-S_2_

MPx:C64S-S_2_ and Prx:C84S-S_2_ were used to measure activity of CgAhp reducing intramolecular disulfide bond (Chen et al. 2021). Oxidized MPx:C64S-S_2_ and Prx:C84S-S_2_ were prepared according to previously described (Li et al. 2021). The kinetic parameters were determined in the presence of varying concentration of MPx:C64S-S_2_ and Prx:C84S-S_2_ (0–25 µM). The enzyme reactions were measured in 50 mM Tris–HCl buffer (pH 7.5), 1 mM EDTA, 500 µM NADH, 5 µM Lpd, 5 µΜ SucB, and 1 µM CgAhp. The assay was performed at 25 °C and the absorption monitored at 340 nm. In this case, the reaction was started by the addition of MPx:C64S-S_2_ and Prx:C84S-S_2_. NADH oxidation was monitored as *A*_340_. The activity was determined after subtracting the spontaneous reduction rate observed in the absence of MPx:C64S-S_2_ or Prx:C84S-S_2_, and the number of micromoles of NADH oxidized per second per micromole of enzyme (i.e. turnover number, s^−1^) was calculated using the molar absorption coefficient of NADH at 340 nm (*Ɛ*_340_) of 6220 M^−1^ cm^−1^. Three independent experiments were performed at each substrate concentration. The *k*_cat_ and *K*_m_ values of CgAhp for MPx:C64S-S_2_ or Prx:C84S-S_2_, were obtained from a non-linear fit with the Michaelis–Menten equation using the program GraphPad Prism 5.

### Insulin disulfide reduction

Insulin disulfide reduction was performed based on the method described by Rosado et al. (2017).

### Peroxidase activity assays

Peroxidase activity were performed by monitoring the decrease in absorbance at 340 nm arising from NADH oxidation (Chen et al. 2021). CgAhp-dependent peroxidase activities of MPx, Prx, Ohr, and OsmC were monitored by linking to the Lpd/SucB/NADH reduced system according to the method of Su et al. (2018). Peroxidase activity was also detected by using the ferrous Xylenol Orange (FOX) assay (Wolff 1994).

### Oxidase activity assay

Oxidase activity was measured as described previously (Rosado et al. 2017).

### Construction of chromosomal fusion reporter strains and β-Galactosidase assay

The *lacZY* fusion reporter plasmid pK18*mobsacB-P*_*cgahp*_::*lacZY* was transformed into WT(pXMJ19), ∆*oasR*(pXMJ19), and ∆*oasR*(pXMJ19-*oasR*) strains by electroporation. The introduced pK18*mobsacB* derivatives were integrated into the chromosome using fusion promoter regions homologous to the genome of *C. glutamicum* by single crossover and then the chromosomal pK18*mobsacB-P*_*cgahp*_::*lacZY* fusion reporter strain was selected by plating on LB agar plates containing 40 µg ml^−1^ NAL, 25 µg ml^−1^ KAN, and 10 µg ml^−1^ CHL (Shen et al. 2005). The resulted strains were grown in LB medium to an optical density at 600 nm of 0.6–0.7 and then treated with different reagents of various concentrations at 30 °C for 30 min. β-galactosidase activities were assayed with O-nitrophenyl-β-D-galactopyranoside (ONPG) as the substrate (Miller 1992). All β-Galactosidase experiments were performed with at least three independent biological replicates.

### Quantitative real-time polymerase chain reaction (qRT-PCR) analysis

Total RNA was isolated from exponentially growing WT(pXMJ19), ∆*oasR*(pXMJ19) and ∆*oasR*(pXMJ19-*oasR*) strains exposed to CHP or *t*-BHP of indicated concentrations for 30 min using the RNeasy Mini Kit (Qiagen, Hilden, Germany) along with the DNase I Kit (Sigma-Aldrich, Taufkirchen, Germany). Purified RNA was reverse-transcribed with random 9-mer primers and MLV reverse transcriptase (TaKaRa, Dalian, China). Quantitative RT-PCR analysis (7500 Fast Real-Time PCR; Applied Biosystems, Foster City, CA) was performed as described previously (Su et al. 2018). The primers used were listed in Supplementary Table S2. To obtain standardization of results, the relative abundance of 16S rRNA was used as the internal standard.

### Electrophoretic mobility shift assay (EMSA)

The binding of OasR to *cgahp* promoters was performed using the method of Su et al. (2018). Briefly, 220-bp *cgahp* DNA promoter (*P*_*cgahp*_) containing the predicted OasR binding site was amplified from the sequence [− 220 to − 1 relative to the ATG start codon of the first open reading frame (ORF) of the *cgahp* gene] using primer pair Ecgahp-F/Ecgahp-R (Supplementary Table S2). Different concentration of purified His_6_-OasR (0–5.0 µg) was incubated with 40 nM *P*_*cgahp*_ in a total volume of 20 µl. A 220-bp fragment from the *cgahp* coding region amplified with primers Control-F and Control-R instead of *P*_*cgahp*_ and bovine serum albumin (BSA) instead of OasR were used as negative controls. The binding reaction buffer contained 10 mM Tris–HCl (pH 7.4), 5 mM MgCl_2_, 50 mM KCl, 5% glycerol, 0.1% Nonidet P 40 (NP40), 1 µg poly(dI:dC), 1 mM dithiothreitol (DTT). The binding reaction mixtures were incubated at room temperature for 30 min and then loaded onto 8% native polyacrylamide gel made with 10 mM Tris buffer containing 50 mM KCl, 5 mM MgCl_2_ and 10% glycero1 in 0.5 × TBE electrophoresis buffer [50 mM Tris, 41.5 mM borate (pH 8.0), 10 mM Na_2_EDTA·H_2_O]. Electrophoresis was performed at 4 °C temperature and 100 V using 1×TBE (89 mM Tris, 89 mM borate, 2 mM EDTA) as the electrophoresis buffer. The gel was subsequently stained with a 10,000-fold diluted SYBR Gold nucleic acid staining solution (Molecular Probes) for 30 min. The DNA bands were visualized with UV light at 254 nm. Experiments were performed with at least three independent biological replicates.

### Statistical analysis

Statistical significance was calculated using one-way ANOVA Dunnett’s multiple comparison test by GraphPad Prism 5 Software (San Diego, California, USA).

## Results and discussion

### Characteristic of CgAhp

The *ncg10877* gene of *C. glutamicum* is located at bp 969458 to 969940, encoding a putative alkyl hydroperoxide reductase (Ahp) of 160 amino acid residues with a molecular mass of 18.0 kDa. The NCgl0877 protein shared amino acid sequence identities of 51.3%, 53.9%, 52.9%, 50%, and 52.4% with the Cys-X-X-Cys active site motif-containing Ahp of *Achromobacter xylosoxidans* (ADP19073), *Nitrosococcus watsonii* (ADJ28176), *Citrobacter freundii* (AUV27934), *Simplicispira suum* (AVO42213), and *Candidatus nitrosoglobus* (BAW80394), respectively (Supplementary Fig. S1A). Ahp proteins were classified into two classes on the basis of functional characterization so far: disulfide oxidoreductases including 2-Cys AhpD and 1-Cys AhpF, and Prx of the thiol-based peroxidase family including AhpC and AhpE (Tartaglia et al. 1990; Wood et al. 2004). According to amino acid sequence, we found that NCgl0877 of *C. glutamicum* shared a highly conserved Cys-X-X-Cys (C-X-X-C) catalytic signature motif with 2-Cys AhpD, which was not the same as that of peroxide-degrading peroxidase with two active cysteines, such as AhpC (Supplementary Fig. S1B). Two-cysteine (2-Cys) AhpC metabolized peroxides via a conserved NH_2_-terminal cysteine residue (C_p_), which undergoes oxidation. To complete the catalytic cycle, the Cys residue must be reduced via a *C*-terminal cysteine residue (C_R_), which was far from C_p_, to result in the formation of an intermolecular disulfide bond in AhpC (Bryk et al. 2000; Zhang et al. 2019). 2-Cys AhpD restored oxidized peroxidases to their reduced form by the *N*- and *C*-terminal Cys of the Cys-X-X-Cys active site motif (Su et al. 2019). Therefore, we speculated that NCgl0877 of *C. glutamicum* may be act as oxidized peroxidase-reducing disulfide oxidoreductase but not peroxidase.

According to the catalytic CXXC motif, oxidized peroxidase-reducing disulfide oxidoreductases reported in *C. glutamicum* so far were classified into six different groups: Trx (Cys-Gly-Pro-Cys (C-G-P-C), Mrx1(Cys-Pro-Tyr-Cys (C-P-Y-C), AhpD (Cys-Gly-Thr-Cys (C-G-T-C) and Cys-Val-Tyr-Cys (C-V-Y-C), DsbA-like Mrx1 (Cys-Pro-Phe-Cys (C-P-F-C), NrdH (Cys-Val-Gln-Cys (C-V-Q-C), and Mrx3 (Cys-Gly-Ser-Cys (C-G-S-C) (Supplementary Fig. S1B). Strikingly, NCgl0877 of *C. glutamicum* formed a new group, preserving the Cys-Pro-Gly-Cys (C-P-G-C) active-site sequence motif. Moreover, the intervening residues between two cysteines in the catalytic CXXC motif of NCgl0877 were more similar to those of Mrx1 (C-P-Y-C) and DsbA-like Mrx1 (C-P-F-C), but was different from those of AhpD (C-G-T-C or C-V-Y-C) (Supplementary Fig. S1B). Previous studies showed that the difference of the intervening residues between two cysteines in the catalytic CXXC motif caused disulfide oxidoreductases to have different enzymatic rates and substrate preference properties (Rosado et al. 2017). Therefore, the discovery of new C-P-G-C active site motif prompted us to investigate the role of *C. glutamicum* NCgl0877.

### *Cgahp* null mutant was sensitive to organic peroxide stress

Recently, Si et al. (2020) found that the *ncgl0877* geneof *C. glutamicum* (designated *cgahp*) was one of the main targets of OasR, which was strongly linked to the oxidative stress response in *C. glutamicum*. Therefore, we speculated that CgAhp may also play a role in oxidative stress response. To gain the physiologically functional insights into the oxidative stress resistance of CgAhp, we investigated the phenotype of a *cgahp* null mutant in *C. glutamicum* RES167 strain, obtained by homologous recombination-based gene knock-out, with regards to ROS resistance by an agar-based disc diffusion assay. As shown in Supplementary Fig. S2, although CgAhp was viewed as non-essential in *C. glutamicum* RES167 under normal growth conditions, the Δ*cgahp*(pXMJ19) strain (the mutant lacking *cgahp* with the empty plasmid pXMJ19) resulted in decreased tolerance to CHP and *t*-BHP as compared with WT(pXMJ19) strain (the wild-type *C. glutamicum* strain with the empty plasmid pXMJ19), giving a significantly larger inhibition zone than WT(pXMJ19) strain. To confirm that the sensitivity to reagents was owing to the absence of *cgahp* gene, the complementary strain Δ*cgahp*(pXMJ19-*cgahp*) was constructed by the introduction of plasmid pXMJ19 *in trans* containing the wild-type *C. glutamicum cgahp* gene into Δ*cgahp* null mutant and complementation experiments were carried out. As shown in Supplementary Fig. S2B, the complementary strain Δ*cgahp*(pXMJ19-*cgahp*) produced significantly smaller inhibition zone under CHP and *t*-BHP again, which were the equivalent of those of the WT(pXMJ19) strains, indicated that resistant phenotypes were almost fully restored in Δ*cgahp*(pXMJ19-*cgahp*) strains. However, there was no significant difference between the WT(pXMJ19), Δ*cgahp*(pXMJ19), Δ*cgahp*(pXMJ19-*cgahp*) strains upon H_2_O_2_, HClO, diamide, CDNB, IAM, STR, CIP, CdCl_2_, and NiSO_4_ challenge. A recent report showed that the lack of *oasR* gene only resulted in increased resistance to organic peroxides (OPs) and the *oasR* expression was induced by OPs but not by other oxidants (Si et al. 2020). Moreover, disulfide oxidoreductases Mrx1, Trx and NCgl0018 in *C. glutamicum* was involved in inorganic oxides, alkylation agents, heavy metal resistance (Li et al. 2021; Che et al. 2020; Chen et al. 2021). These results, combined with up-regulated expression of *cgahp* in Δ*oasR* (Si et al. 2020), indicated that OasR specifically responded to OPs to release the inhibition of *cgahp*, thereby leading to OPs resistance. This phenomenon enabled cells to initiate specific detoxification pathways and respond quickly to environmental pressures. Therefore, CgAhp was involved in OPs stress resistance.

### Formation of an intramolecular disulfide bond Cys42-Cys45 under oxidative stress

**Fig. 1:**
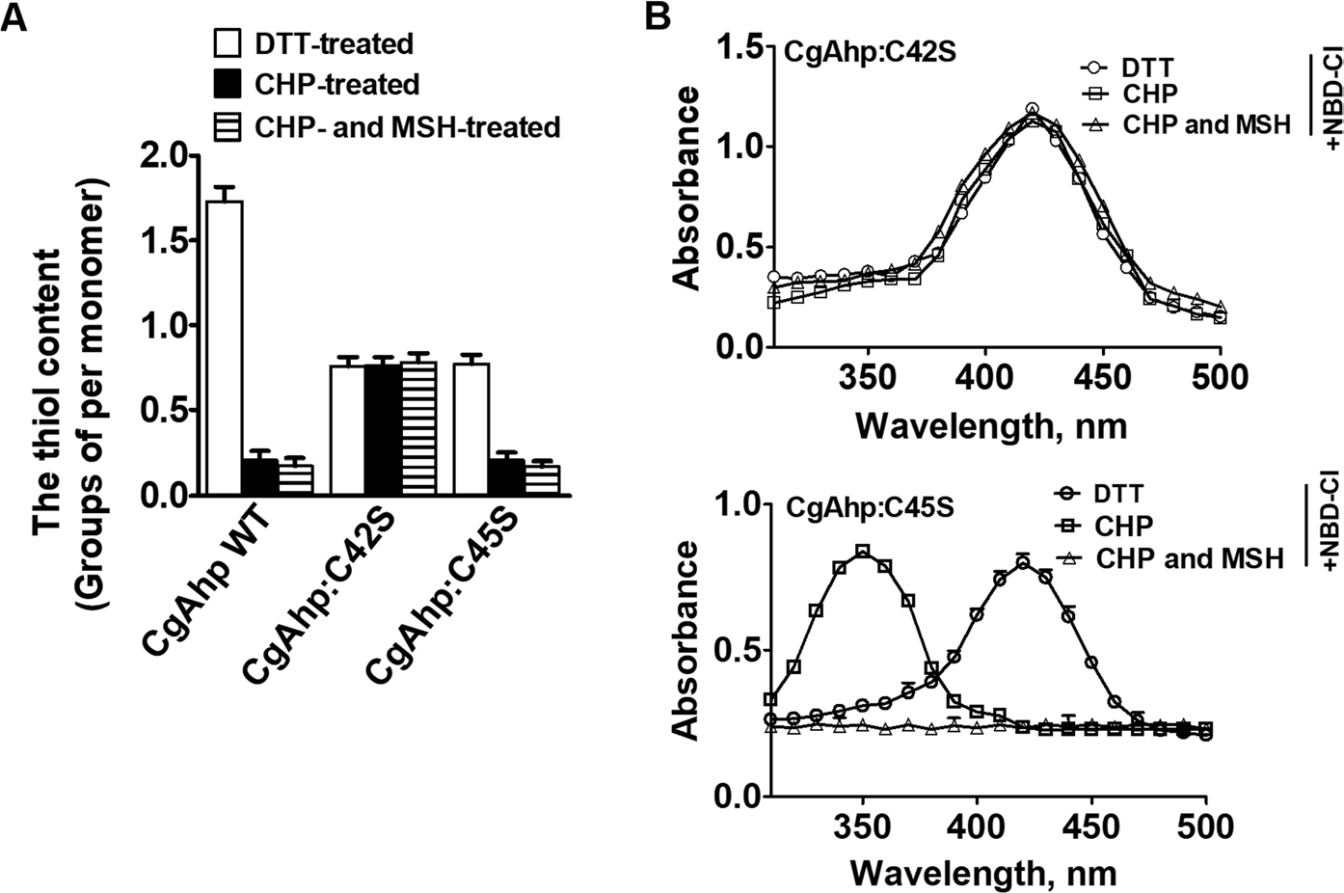
The form and thiol content of DTT-, CHP, or CHP- and MSH-treated CgAhp. **A** Free sulfhydryl groups in CgAhp WT, CgAhp:C42S, and CgAhp:C45S were determined using 5,5′-dithio-bis(2-nitrobenzoicacid) (DTNB). The data were represented as mean ± SD of three independent experiments. **B** Spectrophotometric analysis of NBD-labelled CgAhp:C42S and CgAhp:C45S. Reduced proteins treated with and without CHP or CHP and MSH were modified with NBD-Cl for 30 min. The resulting proteins were analyzed spectrophotometrically at 200–600 nm. The data were represented as mean ± SD of three independent experiments

CgAhp contains a conserved catalytic motif at position 42–45 consisting of C^42^-P-G-C^45^ (Supplementary Fig. S1). Sequence alignment indicates that Cys42 might be the nucleophilic cysteine residue, while Cys45 might be the resolving Cys residue (Supplementary Fig. S1). Therefore, we speculated that Cys42 and Cys45 might participate in the formation of disulfide bonds. To confirm this speculation, we mutated the first and the second cysteine of the CXXC motif to serine to gain two variants of CgAhp, namely, CgAhp:C42S and CgAhp:C45S. CgAhp WT and these two variants of CgAhp with and without CHP treatment were used to perform DTNB analysis and NBD-Cl modification. As shown in Fig. 1A, the DTT-treated CgAhp WT contained 1.73 ± 0.45 thiol groups per monomer, but the thiol content decreased to 0.21 ± 0.12 when CgAhp WT was treated with CHP. The difference of 1.51 thiol groups between the two preparations was linked to the fully oxidation of CgAhp WT after CHP treatment.

NBD-Cl can specifically react with free sulfhydryl groups of cysteines and cysteine sulfenic acid, but not with cysteines that are present as sulfinic acid or sulfonic acid. The covalent attachment of NBD-Cl generated an absorption peak at ∼420 nm upon reaction with thiol groups, whereas it peaked at ∼347 nm upon reaction with sulfenic acids (Baker and Poole 2003). Following the reaction with NBD-Cl, the absorption spectra of CgAhp:C42S variants were unchanged before and after exposure to CHP or both CHP and MSH, exhibiting only the 420 nm peak (Fig. 1B). The CgAhp:C42S variant showed one thiol per monomer before and after CHP or both CHP and MSH treatment, indicating that Cys45 was still in thiol form under exposure to CHP (Fig. 1A). However, CgAhp:C45S under CHP treatment lost one thiol group, compared to the thiol content of DTT-treated state, indicating that Cys42 did not exist as a thiol in CHP-treated CgAhp:C45S variant (Fig. 1B). The redox state of thiol in CgAhp:C45S was further examined using NDB-Cl modification. Non-CHP-treated CgAhp:C45S modified with NBD-Cl produced a new covalently attached spectral species with a λ_max_ of 420 nm, consistent with previously characterized thiol adducts with NBD-Cl (Cys-S-NBD). CHP-treated and NBD-labeled CgAhp:C45S had an absorbance maximum (λ_max_) of 347 nm, representing the NBD-modified product Cys-S(O)-NBD (Ellis and Poole 1997), which clearly signified the detection and trapping of approximately stoichiometric amounts of SOH at Cys42, the only Cys in this variant (Fig. 1B). However, no NBD-Cl labelling peaks occurred in CHP- and MSH-treated CgAhp:C45S proteins. Previous studies showed that MSH reacted with sulfenic acid (Cys-SOH) to form Cys-SSM (Chen et al. 2021; Li et al. 2021). Cys-SSM did not react with NBD-Cl. Therefore, combined with our result that Cys42 was oxidized to form a sulfenic acid (Cys42-SOH), we speculated that *S*-mycothiolation occurred on Cys42 of CgAhp in the presence of MSH and CHP.

### The p*Ka* of the cysteine residues

Previous research has shown the p*K*a of the nucleophilic cysteine involved in the reaction was a determining factor for the rates of the thiol-disulfide exchange reactions (Jensen et al. 2014). The nucleophilic cysteine in the CXXC motif of oxidoreductases was often in the local electrostatic environment due to the influence of nearby residues (Hansen et al. 2005), leading to the phenomenon that the p*K*a value of the *N*-terminal cysteine in the CXXC motif was lower than that of cysteine (8.6) (Lillig et al. 2008). As the low p*K*a value of the nucleophilic cysteine, the *N*-terminal cysteine could perform a nucleophilic attack on the substrate disulphide (Lillig et al. 2008). Thus, the p*K*a of active site residues in CgAhp was determined by recording the absorption at 240 nm during a pH titration (Roos et al. 2013), since the thiolate ion has a higher absorption at this wavelength than the thiol group. As shown in Supplementary Fig. S3, the p*K*a values of the nucleophilic Cys42 and the resolving Cys45 were less than 6 and 8.39, respectively. The result indicated that the low p*K*a value made Cys42 function as the nucleophilic Cys. Moreover, the p*Ka* of the Cys45 (8.39) was already lower than the p*Ka* of the MSH sulfur (8.76) (Sharma et al. 2016), which made Cys45 more attack Cys42-SSM mixed disulfide, leading to the formation of a Cys42-Cys45 disulfide. Together, the above results indicated that Cys45 resolved the mixed disulfide Cys42-SSM or Cys42-SOH, leading to the formation of a Cys42-Cys45 disulfide. The result was consistent with the result of Chen et al. (2021) reported for *C. glutamicum* NCgl0018.

### Oxidized CgAhp (CgAhp-S_2_) was preferentially reduced with electrons from the Lpd/SucB/NADH pathway

Since *C. glutamicum* used three ubiquitous electron transfer pathways, i.e. the MSH/Mtr/NADPH system, TrxR/NADPH system, and Lpd/SucB/NADH system, to reduce disulphide bonds between the active site cysteines in oxidoreductase, we identified possible electron donor pathways coupled to oxidized CgAhp reduction. To do so, CgAhp was first oxidized with a 10-fold molar excess of diamide to obtain CgAhp-S_2_ with a single disulfide bond between its active site cysteines (CgAhp_ox_).

Next, CgAhp-S_2_ was added as a substrate for the electron transfer pathways mentioned above. By monitoring the decrease in the absorption at 340 nm due to NADPH or NADH consumption, we found that oxidized CgAhp-S_2_ obviously obtained electrons transferred by the Lpd/SucB/NADH pathway, while slight electron transfer was observed when the MSH/Mtr/NADPH or TrxR/NADPH electron transfer pathway was used (Supplementary Figs. S4 A–C). Further, we determined steady-state kinetics. As shown in Supplementary Figs. S4 D–F, the *K*_m_ value, *k*_cat_ value, and catalytic coefficient of CgAhp-S_2_ for the MSH/Mtr/NADPH, TrxR/NADPH, or Lpd/SucB/NADH electron donor pathway were calculated to be 12.51 ± 2.37 µM, 0.03 ± 0.002 s^−1^, and 2.39 ± 0.08 × 10^3^ M^−1^ s^−1^; 4.85 ± 0.89 µM, 0.11 ± 0.01s^−1^, and 2.27 ± 0.13 × 10^4^ M^−1^ s^−1^, or 1.21 ± 0.13 µM, 19.61 ± 0.39 s^−1^, and 1.63 ± 0.04 × 10^7^ M^−1^ s^−1^, respectively. It is worth noting that although CgAhp-S_2_ could be reduced by the three electron pathways, the catalytic coefficient of CgAhp-S_2_ with the Lpd/SucB/NADH pathway was several orders of magnitude higher than those with the TrxR/NADPH and MSH/Mtr/NADPH pathways, indicating CgAhp-S_2_ preferred the Lpd/SucB/NADH pathway. So, CgAhp-S_2_ seemed to be mainly reduced by the Lpd/SucB/NADH pathway in *C. glutamicum* but not the TrxR/NADPH and MSH/Mtr/NADPH reducing systems, in line with the result of Su et al. (2019) reported for *C. glutamicum* AhpDs.

#### CgAhp functioned as a weak peroxidase but not oxidase

**Fig. 2:**
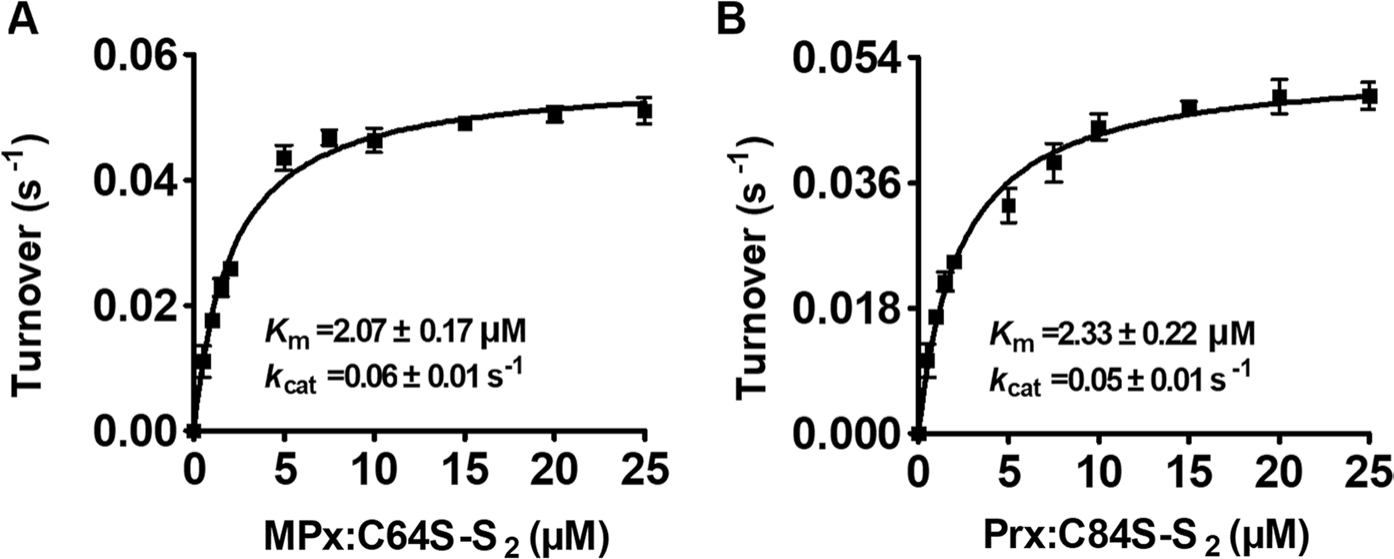
CgAhp reduced disulfide bond of oxidized peroxidase. MPx:C64S-S_2_ (**A**) and Prx:C84S-S_2_ (**B**) could be regenerated by CgAhp electron transfer pathway in vitro. The data were represented as mean ± SD of three independent experiments. The CgAhp reduction of the disulfide bond of MPx:C64S-S_2_ and Prx:C84S-S_2_ followed Michaelis–Menten steady-state kinetics. Different concentrations of oxidized MPx:C64S-S_2_ and Prx:C84S-S_2_ were mixed with a pre-incubated mixture of CgAhp, Lpd, SucB, and NADH

As CgAhp played an important role in the resistance to OPs stresses, we therefore examined the possible role of CgAhp as a peroxidase. H_2_O_2_ and CHP were added to the Lpd/SucB/NADH pathway in the presence and absence of CgAhp, and the ability of CgAhp to reduce H_2_O_2_ and CHP was investigated by following the absorption decrease of NADH at 340 nm (Fig. 2A, B). A very weak peroxidase activity of CgAhp for CHP was observed, as the addition of CgAhp to the reaction mixture containing CHP resulted in a slight increase of NADH consumption at 340 nm. No peroxidase activity of CgAhp for H_2_O_2_ was detected. The peroxidase activity of CgAhp was further corroborated by monitoring the consumption of H_2_O_2_ and CHP in a Fox assay (Fig. 2C, D), indicating that CgAhp by itself showed minimal peroxidatic activity by linking to the Lpd/SucB/NADH pathway, as reported (Bryk et al. 2002). Thus, the result showed that CgAhp was not thiol-dependent alkyl peroxidases.

To investigate its putative DsbA-oxidoreductase activity, we used *E. coli* RNase I as a substrate. RNase I was active with its four disulfide bonds correctly formed, making it an ideal model enzyme for oxidative protein folding evaluation (Messens et al. 2007). We used methylene blue intercalated RNA as a substrate to check the oxidase activity at 659 nm after the incubation of reduced unfolded RNase I with CgAhp (Greiner-Stoeffele et al. 1996). CgAhp did not have a capacity of catalyzing the oxidative refolding of RNase I (Fig. 2E). Reduced RNase I (unfolded) demonstrated 14.6% of activity relative to folded RNase I (100%). In contrast, in the presence of *E. coli* DsbA, which has been proven to be an oxidase, 67.7% of activity was recovered (Rosado et al. 2017). Thus, CgAhp did not act as an oxidase.

### CgAhp behaved like *C. glutamicum* AhpD in regenerating thiol-dependent peroxidase coupled to the Lpd/SucB/NADH electron pathway

To determine whether CgAhp was able to regenerate thiol-dependent peroxidases, the catalytic constants of peroxidase with the CgAhp/Lpd/SucB/NADH system as the recycling reductants were determined under steady-state conditions at saturating concentrations of peroxides and different concentrations of the recycling reductant CgAhp (0 to 500 µM). As shown in Table 1, the *k*_*cat*_ and *K*_*m*_ values of MPx for CHP with the CgAhp/Lpd/SucB/NADH system were 4.11 ± 0.53 s^−1^ and 28.92 ± 0.31 µM, respectively. This corresponded to catalytic efficiencies of 14.27 × 10^4^ M^−1^ s^−1^, in accordance with data obtained on AhpD from *C. glutamicum* (AhpD2, around 34.7 × 10^4^ M^−1^ s^−1^) (Su et al. 2019). Similar results were also observed in Prx, Ohr, and OsmC with the CgAhp/Lpd/SucB/NADH system as the terminal electron acceptor for CHP elimination. The catalytic efficiencies of Prx, Ohr, and OsmC for CHP with the CgAhp/Lpd/SucB/NADH system were 9.3 × 10^4^ M^−1^ s^−1^, 106.9 × 10^4^ M^−1^ s^−1^, and 186.3 × 10^4^ M^−1^ s^−1^, respectively. Although the CgAhp/Lpd/SucB/NADH system supported the peroxidase activities of MPx and Prx when H_2_O_2_ was used as substrate, it showed comparably low activity. Of note, the CgAhp/Lpd/SucB/NADH reducing system facilitated Ohr and OsmC activity very poorly when H_2_O_2_ was used as substrate, in line with the results of Si et al. (2015a; 2019) reported for only and mainly organic peroxide-detoxifying Ohr and OsmC in *C. glutamicum*, respectively. Previous studies showed that Ohr and OsmC could employ Trx regeneration systems in reducing CHP substrate (the catalytic efficiencies of Ohr and OsmC were 10 × 10^4^ and 21.2 × 10^4^, respectively), they had higher affinity for CgAhp than Trx in vitro. These data indicated that the CgAhp/Lpd/SucB/NADH system was more efficient in supporting the peroxidase activity of Ohr and OsmC when CHP was used as substrate but not H_2_O_2_. Moreover, CgAhp preferentially supported the peroxidase activity of Ohr and OsmC. When CHP was used as substrate, the catalytic efficiencies of MPx and Prx with the CgAhp/Lpd/SucB/NADH system was significantly lower than data obtained on *C. glutamicum* MPx and Prx with the Trx system (MPx, 58.5 × 10^4^ M^−1^ s^−1^; Prx, 264.1 × 10^4^ M^−1^ s^−1^) (Si et al. 2015b, 2017), and about 8–20 times lower than those of the *C. glutamicum* Ohr and OsmC-catalyzed reaction with the CgAhp/Lpd/SucB/NADH system. In addition, the catalytic coefficients of MPx and Prx with the CgAhp/MSH/Mtr/NADPH and CgAhp/TrxR/NADPH system as the recycling reductants were detected. As shown in Table 1, although CgAhp could support the peroxidase activity of MPx and Prx linked to MSH/Mtr/NADPH or TrxR/NADPH systems, their catalytic coefficients were only at a low rate of 3–70 M^−1^ s^−1^, several orders of magnitude lower than those with the Lpd/SucB/NADH pathway, indicating that CgAhp linked to the CgAhp/MSH/Mtr/NADPH or CgAhp/TrxR/NADPH system was highly unlikely in vivo.

**Table 1:** The CgAhp acted as a thiol-dependent peroxidase reducing power

Peroxidase	CHP			H_2_O_2_		
	*K*_m_ (µM)	*k*_cat_ (s^−1^)	*k*_cat_/*K*_m_ × 10^4^ (M^−1^ s^−1^)	*K*_m_ (µM)	*k*_cat_ (s^−1^)	*k*_cat_/*K*_m_ × 10^4^ (M^−1^ s^−1^)
CgAhp/Lpd/SucB/NADH						
MPx	28.92 ± 0.31	4.11 ± 0.53	14.27	44.73 ± 0.52	2.94 ± 0.55	6.53
Prx	52.74 ± 3.41	4.93 ± 0.45	9.32	99.63 ± 2.25	1.51 ± 0.22	1.53
Ohr	7.36 ± 0.94	7.83 ± 0.42	106.93	ND	ND	ND
OsmC	5.17 ± 0.42	9.56 ± 0.34	186.32	ND	ND	ND
CgAhp/MSH/Mtr/NADPH						
MPx	823.46 ± 82.52	0.02 ± 0.003	0.002	919.81 ± 56.31	0.005 ± 0.02	0.001
Prx	898.37 ± 45. 47	0.01 ± 0.004	0.001	965.63 ± 74.06	0.003 ± 0.01	0.0003
Ohr	763.91 ± 35.27	0.03 ± 0.005	0.004	ND	ND	ND
OsmC	806.94 ± 54. 86	0.02 ± 0.007	0.002	ND	ND	ND
CgAhp/TrxR/NADPH						
MPx	703.91 ± 40.56	0.04 ± 0.02	0.006	853.41 ± 63.25	0.007 ± 0.01	0.001
Prx	818.37 ± 28.87	0.01 ± 0.03	0.001	898.37 ± 91.59	0.004 ± 0.01	0.0004
Ohr	695.34 ± 55.31	0.05 ± 0.03	0.007	ND	ND	ND
OsmC	772.83 ± 36.78	0.03 ± 0.06	0.004	ND	ND	ND

Peroxidase assays were performed as described in experimental procedures with peroxides (2 mM) and peroxidase (1 µM) using the CgAhp/Lpd/SucB/NADH system (0–500 µM CgAhp, 5 µM Lpd, 5 µM SucB, and 500 µM NADH), CgAhp/MSH/Mtr/NADPH system (0–500 µM CgAhp, 5 µM Mtr, 500 µM MSH, and 500 µM NADPH), or CgAhp/TrxR/NADPH system (0–500 µM CgAhp, 5 µM TrxR, and 500 µM NADPH). The data were presented as means of values obtained from three independent assays and analyzed by non-linear regression using the program GraphPad Prism 5

*CHP* cumene hydroperoxide, *H*_2_*O*_2_ hydrogen peroxide, *ND* not detectable under the conditions used

All together, these results indicated that (i) CgAhp preferably produced a robust, NADH-dependent, peroxidase activity of Ohr and OsmC; (ii) CgAhp was an important cytoplasmic alkyl hydroperoxide oxidoreductase involved in regeneration of oxidized peroxidase; (iii) CgAhp was linked to the Lpd/SucB/NADH pathway.

### Disulfide bond reduction by CgAhp using electrons from the Lpd/SucB/NADH pathway

**Fig. 3:**
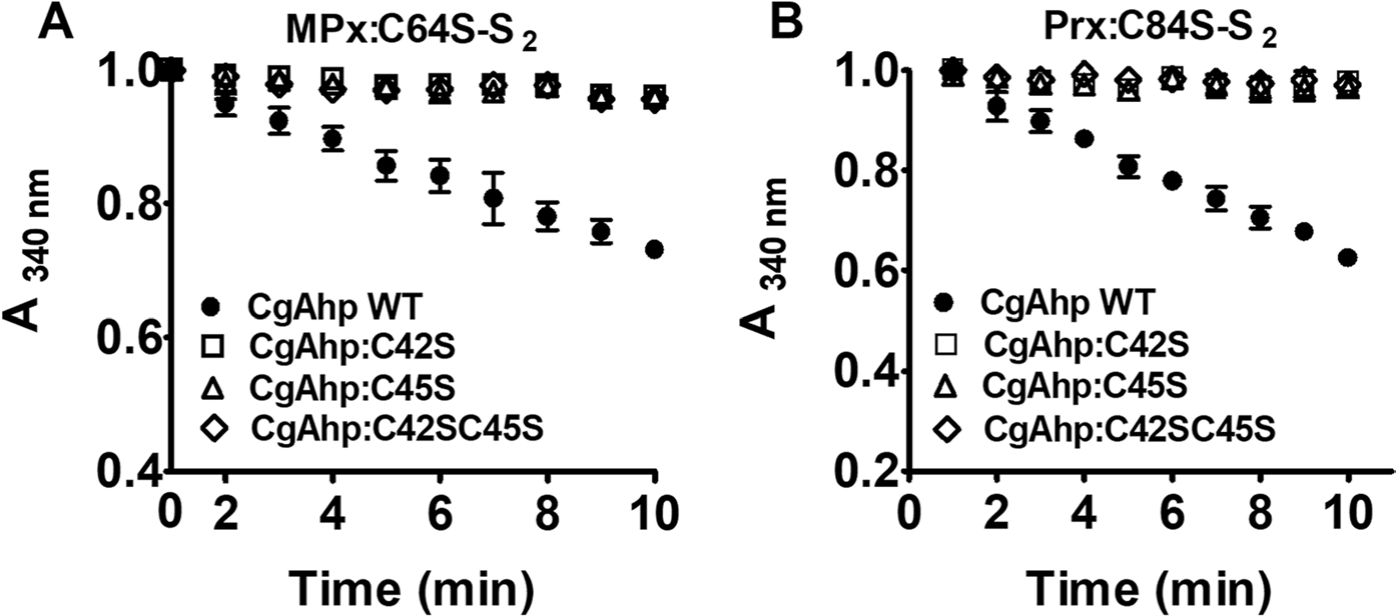
CgAhp reduced disulfide bond via a dithiol mechanism. MPx:C64S-S_2_ (**A**) and Prx:C84S-S_2_ (**B**) could be regenerated by CgAhp WT electron transfer pathway in vitro. Oxidized MPx:C64S-S_2_ and Prx:C84S-S_2_ were mixed with a pre-incubated mixture of CgAhp (WT, CgAhp:C42S, or CgAhp:C45S), Lpd, SucB, and NADH. The decrease in *A*_340nm_, due to NADH oxidation, was monitored in function of time

Peroxidase MPx and Prx metabolizing peroxides in vivo could form two different states of intramolecular disulfide bond (S-S) and the protein-MSH mixed disulfide (Si et al. 2015b, 2017). CgAhp, supporting the peroxidase activity of MPx and Prx, had C-P-X-C motif that was similar to that of Mrx1 and DsbA-like Mrx1 (Supplementary Fig. S1). Therefore, we investigated the possible role of CgAhp in reducing the intramolecular disulfide (S–S) and mixed disulfide of peroxidase in a coupled Lpd/SucB/NADH pathway. Because once determined that Cys64 of MPx and Cys84 of Prx had no direct function in the catalytic mechanism and that Cys79 of MPx and Cys97 of Prx formed a disulfide with Cys36 of MPx and Cys63 of Prx at the end of the catalytic cycle, respectively, we decided to use MPx:C64S and Prx:C84S to study the follow experiments (Si et al. 2015b, 2017). We employed in vitro assay system (see “Materials and Methods”) by using pre-oxidized disulfide bonded MPx:C64S-S_2_ and Prx:C84S-S_2_ as substrates together with *C. glutamicum* Lpd, SucB and NADH. From the Michaelis–Menten kinetic plot, we obtained *k*_cat_ of 0.06 ± 0.01 s^−1^ and 0.05 ± 0.01 s^−1^, and *K*_*m*_ of 2.07 ± 0.17 µM and 2.33 ± 0.22 µM, which result in a specificity constant (*k*_cat_/*K*_m_) of 2.84 × 10^4^ M^−1^s ^−1^ and 2.32 × 10^4^ M^−1^s ^−1^, respectively (Fig. 3A, B). Compared with MPx with *C. glutamicum* Trx (8.43 × 10^4^ M ^−1^ s^−1^) (Pedre et al. 2015), the catalytic efficiency of MPx disulfide reduction with CgAhp is slightly lower. To further assess whether CgAhp possessed general thiol-disulfide redox activity as Trx, we further tested the capacity of recombinant CgAhp to reduce disulfide compound Insulin. Insulin, containing disulfides, was typically used for determining the ability of protein disulfide reduction (Holmgren 1979). We therefore detected the ability of CgAhp/Lpd/SucB/NADH system to reduce insulin. As shown in Table 2, CgAhp was shown to reduce insulin disulfides in the presence of the Lpd/SucB/NADH pathway. Its rate of precipitation was higher than those of Mrx1 (22.67 ± 0.02) and DsbA-like Mrx1 Rv2466c (13.03 ± 0.02) from *M. tuberculosis* (Rosado et al. 2017). These results demonstrated that CgAhp was almost as effective as Trx in reducing disulfide bonds.

**Table 2:** Insulin reduction parameters

Substrates	Control^*a*^	CgAhp
Rate of precipitation (*A*_600_ × 10^− 5^ s^−1^) 4.7 ± 0.3		27.89 ± 3.4
Starting point (s)	2934	1135

^*a*^Control, reaction without catalyst

### CgAhp reduced intramolecular disulfide bond via a dithiol mechanism

**Fig. 4:**
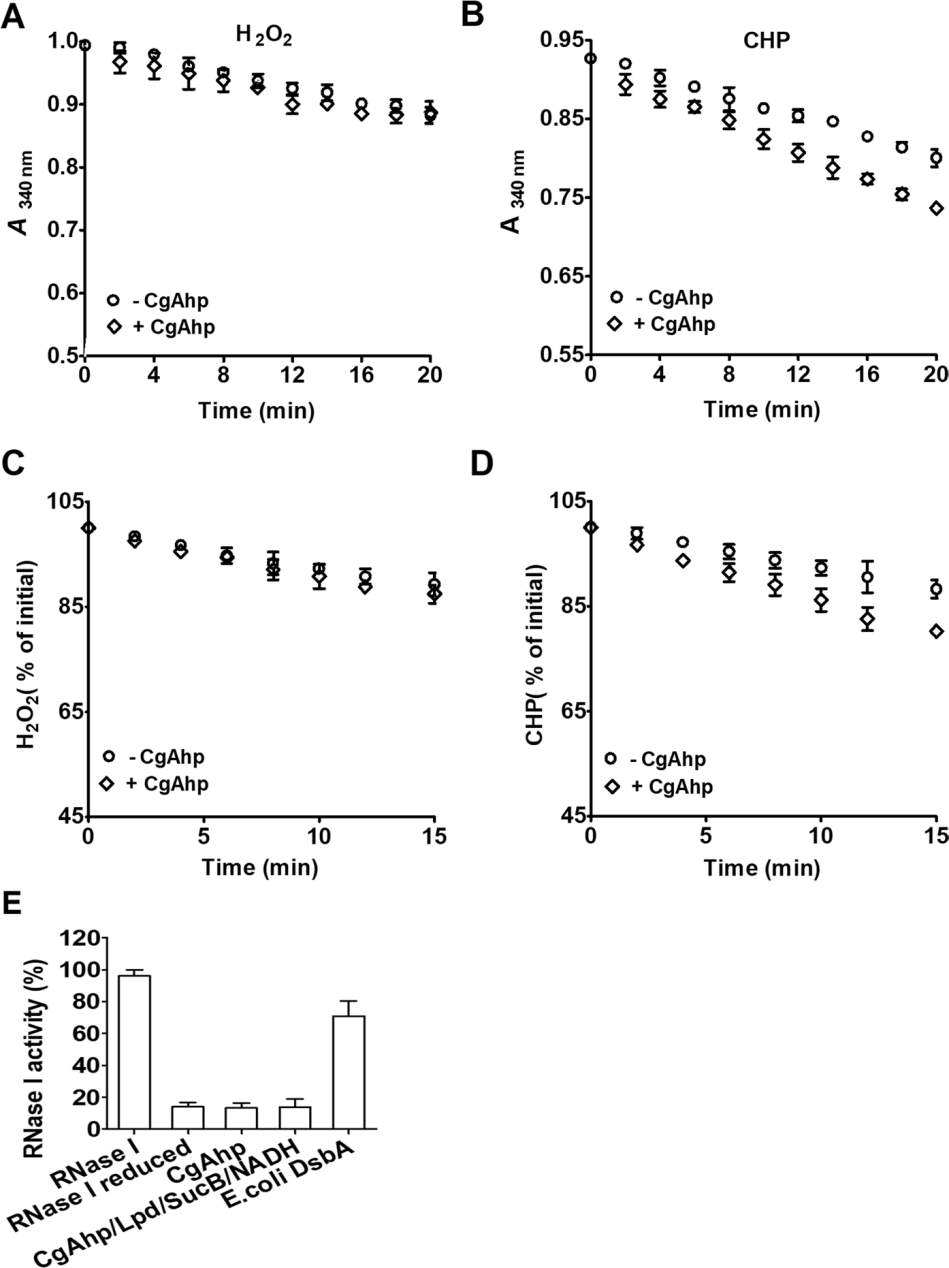
CgAhp was a weak peroxidase but not oxidase. **A** and **B** Peroxidase activities of CgAhp. The coupled assay of the 1 µM CgAhp/3 µM Lpd/3 µM SucB/500 µM NADH pathway with 1mM H_2_O_2_ or and CHP as substrates. Peroxidase activities were determined by recording NADH oxidation at 340 nm. A control reaction in the absence of CgAhp was included. **C** and **D** The FOX assay of the 1 µM CgAhp/3 µM Lpd/3 µM SucB/500 µM NADH pathway with H_2_O_2_ and CHP as substrates. The consumption of H_2_O_2_ and CHP by the FOX assay in function of time was shown. **E** CgAphp was not an oxidase. The methylene blue RNA intercalating assay was used to quantify the activity of RNase I (0.5 µM). *E. coli* RNase I was reduced, and the recovering of activity was detected with 5 µM CgAhp in the presence or absence of 3 µM Lpd/3 µM SucB/500 µM NADH pathway or *E. coli* DsbA. CgAhp did not have an ability of catalyzing the disulfide bond formation in a pre-reduced RNase I. *E. coli* DsbA was used as a positive control

To check whether CgAhp used one active site cysteine or two active site cysteines in the reaction process, we mutated the first, the second, and both cysteines in the catalytic CXXC active site motif of CgAhp to serine, respectively, and we expressed and purified CgAhp:C42S, CgAhp:C45S, and CgAhp:C42SC45S mutants to homogeneity. The functionalities of the CgAhp:C42S, CgAhp:C45S, and CgAhp:C42SC45S to reducing oxidized MPx:C64S-S_2_ and Prx:C84S-S_2_ were tested in progress curves by following the oxidation of NADH in the presence of Lpd and SucB. As shown in Fig. 4A, B, electron transfer was almost the same as back ground levels when CgAhp:C42S, CgAhp:C45S, or CgAhp:C42SC45S was present, indicating that both cysteines in the CXXC motif of CgAhp were essential for transferring electron for peroxidase. Only the sample with oxidized MPx:C64S-S_2_ and Prx:C84S-S_2_ coupled to the CgAhp/Lpd/SucB/NADH electron transfer pathway showed consumption of NADH. Mutants could not replace CgAhp WT. As such, CgAhp was functioning as a dithiol reductase with essential *N*-terminal cysteine and *C*-terminal cysteine.

### OasR negatively regulated CgAhp expression in *C. glutamicum*

**Fig. 5:**
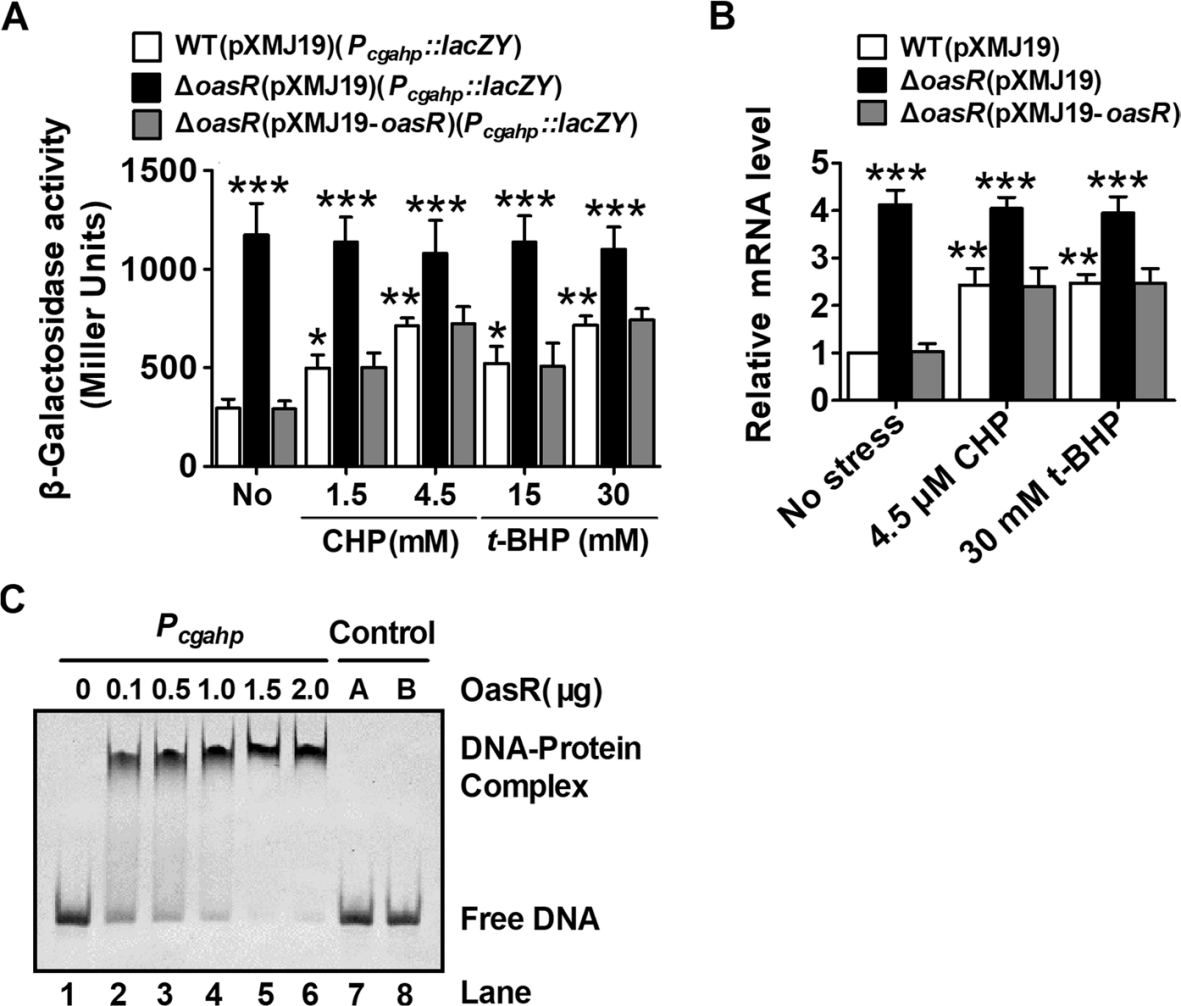
Negative regulation of *cgahp* expression by OasR in *C*. *glutamicum****.*** **A** β-Galactosidase analysis of the *cgahp* promoter activity was performed using the transcriptional *P*_*cgahp*_::*lacZY* chromosomal fusion reporter expressed in indicated strain under different adverse condition for 30 min. Mean values with standard deviations (error bar) from at least three independent biological replicates were shown. The asterisk indicated a significant correlation between the treated and untreated WT(pXMJ19)(*P*_*cgahp*_::*lacZY*) strains or the ∆*oasR*(pXMJ19)(*P*_*cgahp*_::*lacZY*) and untreated WT(pXMJ19)(*P*_*cgahp*_::*lacZY*) strains at ***P ≤ 0.01, **P ≤ 0.01, and *P ≤ 0.05. **B** qRT-PCR assay was performed to analyze the expression of *cgahp*. Exponentially growing *C. glutamicum* cells were exposed to different reagents at indicated concentrations for 30 min. The levels of *cgahp* expression were determined by quantitative RT-PCR. The mRNA levels were presented relative to the value obtained from WT cells without treatment. The values represent the mean results from three independent cultivations, with standard errors.The asterisk indicated a significant correlation between the treated and untreated WT(pXMJ19) strains or the ∆*oasR*(pXMJ19) and untreated WT(pXMJ19) strains at ***P ≤ 0.001 and **P ≤ 0.01. **C** EMSA was performed to analyze the interactions between the *cgahp* promoter (*P*_*cgahp*_) and His_6_-OasR. As negative controls, a 220-bp fragment amplified from the *cgahp* coding region using the primers control F and control R instead of the 220-bp *cgahp* promoter (control A, lane 6) and an irrelevant protein BSA instead of His_6_-OasR (control B, lane 7) were included in the binding assays

The expression of *C. glutamicum* AhpD was induced by oxidative stress (Su et al. 2019). Moreover, *cgahp* mutants exhibited sensitivity to organic peroxide. Therefore, to test whether *cgahp* expression responded to OPs, qRT-PCR at the transcriptional level was performed. Transcriptional and translational *lacZY* genes fused to the probable promoter region of *cgahp* were constructed as described in “Materials and Methods”. The β-galactosidase activity of *P*_*cgahp*_::*lacZY* chromosomal promoter fusion reporter was determined in bacterial cells treated with different concentrations of CHP and *t*-BHP (Fig. 5A). The results showed that the increased levels of β-Galactosidase activity were attributed to *cgahp* promoter in the CHP- and *t*-BHP-induced WT(pXMJ19)(*P*_*cgahp*_::*lacZY*) reporter strains. The enhanced β-Galactosidase activity observed under induction (Fig. 5A) was consistent with the increased mRNA levels in the WT(pXMJ19) strains induced by CHP- and *t*-BHP by qRT-PCR analysis (Fig. 5B). This indicated that CHP- and *t*-BHP induced the expression of *cgahp* in this pathway, thereby increasing the resistance of *C. glutamicum* to OPs conditions.

Recently, Si et al. (2020) found CgAhp was one of the main targets of OasR by microarray analysis, which was strongly linked to the oxidative stress response in *C. glutamicum*. Therefore, we detected OasR’s regulatory capacity for CgAhp. As shown in Fig. 5A, the β-galactosidase activity were approximately four times higher in strain Δ*oasR*(pXMJ19) (strain lacking *oasR* gene contained empty pXMJ19) than in the WT(pXMJ19) strain, indicating that the *cgahp* promoter was negatively regulated by OasR. The negative regulation of *cgahp* by OasR was also confirmed by qRT-PCR, with the observation that the mRNA levels of *cgahp* increased by 4-fold in the mutant Δ*oasR*(pXMJ19) mutant and restored to the wild-type level in the complemented strain ∆*oasR*(pXMJ19-*oasR*) (Fig. 5B). Moreover, transcription of *cgahp* was not increased in the.

Δ*oasR*(pXMJ19) mutant under the CHP- and *t*-BHP-induced conditions. However, *cgahp* transcription increased WT(pXMJ19) strain (Fig. 5A, B). To further determine whether OasR regulated CgAhp expression directly, we examined the interaction between OasR and the CgAhp promoter using EMSA. Incubation of a 220-bp DNA element containing the *cgahp* promoter (*P*_*cgahp*_) sequence (− 220 to − 1 relative to the ATG start codon of the first ORF of the *cgahp* gene) with His_6_-OasR led to the formation of DNA–protein complexes, and the abundance of such complexes depended on the amount of OasR (Fig. 5C left panel). However, both a 220-bp control DNA fragment amplified from the *cgahp* coding open reading frame region and BSA instead of His_6_-OasR showed no detectable binding (Fig. 5C, lane 6 and 7). Thus, OasR directly repressed the expression of *cgahp*.

## Conclusion

In this study, we revealed a novel alkyl hydroperoxide reductase CgAhp by physiological and biochemical analysis. CgAhp enhanced *C. glutamicum* resistance to organic peroxide stress. The physiological roles of CgAhp in resistance to organic peroxide stress were corroborated by its induced expression under stresses, directly regulated by OasR (organic peroxide- and antibiotic- sensing regulator). Unlike classic DsbA, CgAhp displayed no oxidase activity. Moreover, CgAhp by itself showed minimal peroxidatic activity. Although its active-site motif was similar with those of the thiol-disulfide oxidoreductases such as Trx, Mrx1 and AhpD, CgAhp had a similar mode of action as the previously characterized AhpD proteins, displaying very high activity in regenerating thiol-dependent peroxidase. Compared with Trx and Mrx1, CgAhp can use the MSH/Mtr/NADPH, the Trx/TrxR/NADPH and Lpd/SucB/NADH pathways as electrons, but preferred the Lpd/SucB/NADH system. CgAhp used dithiol mechanism to restore various oxidized peroxidases to their reduced forms. CgAhp contained a new C-P-G-C active-site sequence motif, which was more similar to those of Mrx1 and DsbA-like Mrx1 and different from those of AhpDs (C-G-T-C or C-V-Y-C). However, site-directed mutagenesis in combination with enzymatic assays suggested CgAhp was an AhpD-like disulfide oxidoreductase rather than Mrx1-like or DsbA-Mrx1-like oxidoreductase. Based on our results, a catalytic model of CgAhp could be proposed. In the first step, Cys42 reacted with intramolecular disulfide of the target protein, forming an intermediate dithiol bridge between Cys42 and the target protein. For an intermediate dithiol bridge, two reduction pathways could take place. In the presence of MSH, MSH attacked dithiol bridge, releasing reduced target protein and a mycothiolated Cys42. Cys45 nucleophilicly attacked mycothiolated Cys42, releasing MSH and a new Cys42-Cys45 disulfide. In a parallel reaction, for an intermediate dithiol bridge between Cys42 and the target protein, Cys45 solved it to form a Cys42-Cys45 disulfide. Next, Cys42-Cys45 disulfide was reduced by dihydrolipoamide succinyltransferase SucB, lipoamide reductase Lpd, and NADH. Overall, this is the first report demonstrating that CgAhp preserving a new C-P-G-C active-site sequence motif represented a class of AhpD-like molecules that enabled an antioxidant defense in *C. glutamicum* mainly by sustaining peroxidase activity of Ohr and OsmC. This study paved the way for correctly classifying similar enzymes from other organisms and expanded the type of disulfide oxidoreductases.

## Supplementary Information

Below is the link to the electronic supplementary material.

Supplementary material 1 (DOCX 3262.3 kb)

## Declarations

### Confict of interest

The authors declare no compete of interests.
