# Supplementary material for: Characterization of oxidative stress-induced cgahp, a gene coding for alkyl hydroperoxide reductase, from industrial importance Corynebacterium glutamicum
Source: Biotechnol Lett. 2023 Aug 22;45(10):1309–26. doi: 10.1007/s10529-023-03421-8 (PMC10460364; doi:10.1007/s10529-023-03421-8)
Supplement: Supplementary file 1 — Supplementary material 1 (DOCX 3262.3 kb) [file 10529_2023_3421_MOESM1_ESM.docx]

**Supplemental Data**

**Table S1.** Bacterial strains and plasmids used in this study.

| **Strains or plasmids** | **Relevant genotype description** | **References** |
| --- | --- | --- |
| **Strains** | | |
| ***Corynebacterium glutamicum*** | | |
| RES167 | Restriction-deficient mutant of ATCC13032, Δ(*cglIM-cglIR-cglIIR*) | Tauch et al., 2002 |
| Δ*cgahp* | *cgahp* deleted in RES167 | This study |
| Δ*cgahp*(pXMJ19) | Δ*cgahp* containing pXMJ19 vector | This study |
| Δ*cgahp*(pXMJ19-*cgahp*) | Complementation of *cgahp* in Δ*cgahp* | This study |
| WT(pXMJ19) | RES167 containing pXMJ19 vector | This study |
| Δ*oasR*(pXMJ19) |  | Si et al., 2020 |
| Δ*oasR*(pXMJ19-*oasR*) |  | Si et al., 2020 |
| ***E. coli*** | | |
| BL21(DE3) | *E. coli* expression host, *hsdS gal* (*λc*I*ts*857 *ind-l* *Sam7 nin-*5 *lac UV5-*T7 gene 1) | Novagen |
| JM109 | *recA1 supE44 endA1 hsdR17 gyrA96 relA1 thi* Δ(*lac-proAB*)F′(*traD36 proABlacI*^q^ *lacΔZM15*) | Stratagene |
| **Plasmids** | | |
| pK18*mobsacB* | Suicide plasmid carrying *sacB* for selecting double crossover in *C. glutamicum*, Km^r^ | Schäfer et al.,1994 |
| pK18*mobsacB-*Δ*cgahp* | Construct used for in-frame deletion of *cgahp* | This study |
| pK18*mobsacB-P_cgahp_::lacZY* | *P_cgahp_::lacZY* fusion in pK18*mobsacB* | This study |
| pXMJ19 | Shuttle vector (*P_tac_ lacI^q^ pBL1 oriV_C. glutamicum_* pK18 *oriV_E. coli_*) | Jakoby et al.,1999 |
| pXMJ19-*cgahp* | *cgahp* cloned into pXMJ19 for complementation | This study |
| pET28a | Expression vector with N-terminal hexahistidine affinity tag | Novagen |
| pET28a*-cgahp* | *cgahp* in pET28a | This study |
| pET28a-*cgahp:C42S* | *cgahp:C42S* in pET28a | This study |
| pET28a-*cgahp:C45S* | *cgahp:C45S* in pET28a | This study |
| pET28a*-mrx1* |  | Si et al., 2014 |
| pET28a*-mtr* |  | Si et al., 2014 |
| pET28a*-sucB* |  | Si et al., 2015 |
| pET28a*-trx* |  | Si et al., 2014 |
| pET28a*-trxR* |  | Si et al., 2014 |

**References**

Tauch A, Kirchner O, Loffler B, Gotker S, Pühler A, Kalinowski J (2002) Efficient electro transformation of *Corynebacterium diphtheriae* with a mini-replicon derived from the plasmid pGA1. Curr Microbiol 45: 362-367.

Si M, Chen C, Che CC, Liu Y, Li XN, Su T (2020) The thiol oxidation-based sensing and regulation mechanism for the OasR-mediated organic peroxide and antibiotic resistance in *C. glutamicum. Biochem J*  477:3709-3727.

Si MR, Zhang L, Yang ZF, Xu YX, Liu YB, Jiang CY (2014) NrdH Redoxin enhances resistance to multiple oxidative stresses by acting as a peroxidase cofactor in *Corynebacterium glutamicum*. Appl Environ Microbiol 80(5):1750-1762.

Si M, Wang J, Xiao X, Guan J, Zhang Y, Ding W (2015) Ohr Protects *Corynebacterium glutamicum* against Organic Hydroperoxide Induced Oxidative Stress. PLoS One 10(6): e0131634.

Schäfer A, Tauch A, Jager W, Kalinowshi J, Thierbach G, Pühler A (1994) Small mobilizable multi-purpose cloning vectors derived from the *Escherichia coli* plasmids pK18 and pK19: selection of defined deletions in the chromosome of *Corynebacterium glutamicum*. Gene 145:69-73.

Jakoby M, Ngouoto-Nkili CE, Burkovski A (1999) Construction and application of new *Corynebacterium glutamicum* vectors. Biotechnol. Techniques 13:437-441.

**Table S2.** Primers used in this study.

| **Primers** | **5’-3’ sequence** |  |
| --- | --- | --- |
| Ccgahp-F | AGCGGTCGACATGAGTTCGCTCGACAATGCCC (*Sal*I) | For cloning *cgahp* into pXMJ19 |
| Ccgahp-R | CGCGGATCCTTAGGTTTCGTCCGTCTCTGAC (*Bam*HI) |  |
| Ocgahp-F | CGCGGATCCATGAGTTCGCTCGACAATGCCC (*Eco*RI) | For cloning *cgahp* wild type and mutants into pET28a |
| Ocgahp-R | CCGCTCGAGTTAGGTTTCGTCCGTCTCTGAC (*Xho*I) |  |
| Dcgahp-F1 | CAAGAATTCCTTGAGCTCGCAAGAAGTGCTCGCTC (*Eco*RI) | To generate pK18*mobsacB-*Δ*cgahp* |
| Dcgahp-R1 | CCTGAACATCCAATTCCAGCAGC |  |
| Dcgahp-F2 | GCTGCTGGAATTGGATGTTCAGGATTGCTGCTCGGCAGTTTGCTGTC |  |
| Dcgahp*-*R2 | GGAAGATCTAGTGCTGCCATTCCGGTGATGCAC (*Bgl*II) |  |
| Ocgahp-C42S-F | GTGTTTCAGATGCTA*A*GCCCTGGATGCGTG | To generate *cgahp:C42S* DNA fragment |
| Ocgahp-C42S-R | CACGCATCCAGGGC*T*TAGCATCTGAAACACC |  |
| Ocgahp-C45S-F | GCTATGCCCTGGA*A*GCGTGAATCACG | To generate *cgahp:C45S* DNA fragment |
| Ocgahp-C45S-R | CGTGATTCACGC*T*TCCAGGGCATAGC |  |
| *P_cgahp_*-F | TCCCCCGGGATAATCATTGGAGTTTGTTGCAGC (*Sma*I) | To generate pK18*mobsacB-P_cgahp_::lacZY* |
| *P_cgahp_*-R | ACTAGTGAGTTAAACCATGAGTTCGCTC (*Spe*I) |  |
| lacZY-F1 | GAGTTAAACCATGAGTTCGCTCACTAGT ATGACCATGATTACGGATTC(*Spe*I) |  |
| lacZY-R | AAAACTGCAGTTAAGCGACTTCATTCACCTG(*Pst*I) |  |
| Qcgahp-F | CCTGCGCGGCAAGGTTGTGGTGG | RT-PCR |
| Qcgahp-R | ACTTTCAAAGCCTCAGGTGTCATC |  |
| Ecgahp-F | GGTGTTAATTGACCAATCGAAG | To produce the 220-bp EMSA promoter DNA |
| Ecgahp-R | GGTTTAACTCCCTTCATGAAATG |  |
| Control-F | CCTGCGCGGCAAGGTTGTGGTG | To produce the 220-bp EMSA control DNA |
| Control-R | CACTGCCACGGGGAACTTGATC |  |
| 16 S rRNA-F | ACCCTTGTCTTATGTTGCCAG | RT-PCR |
| 16 S rRNA-R | TGTACCGACCATTGTAGCATG |  |

Underlined sites indicated restriction enzyme cutting sites added for cloning. Letters in italic denoted the mutation sites in overlap PCR for site-directed mutation.


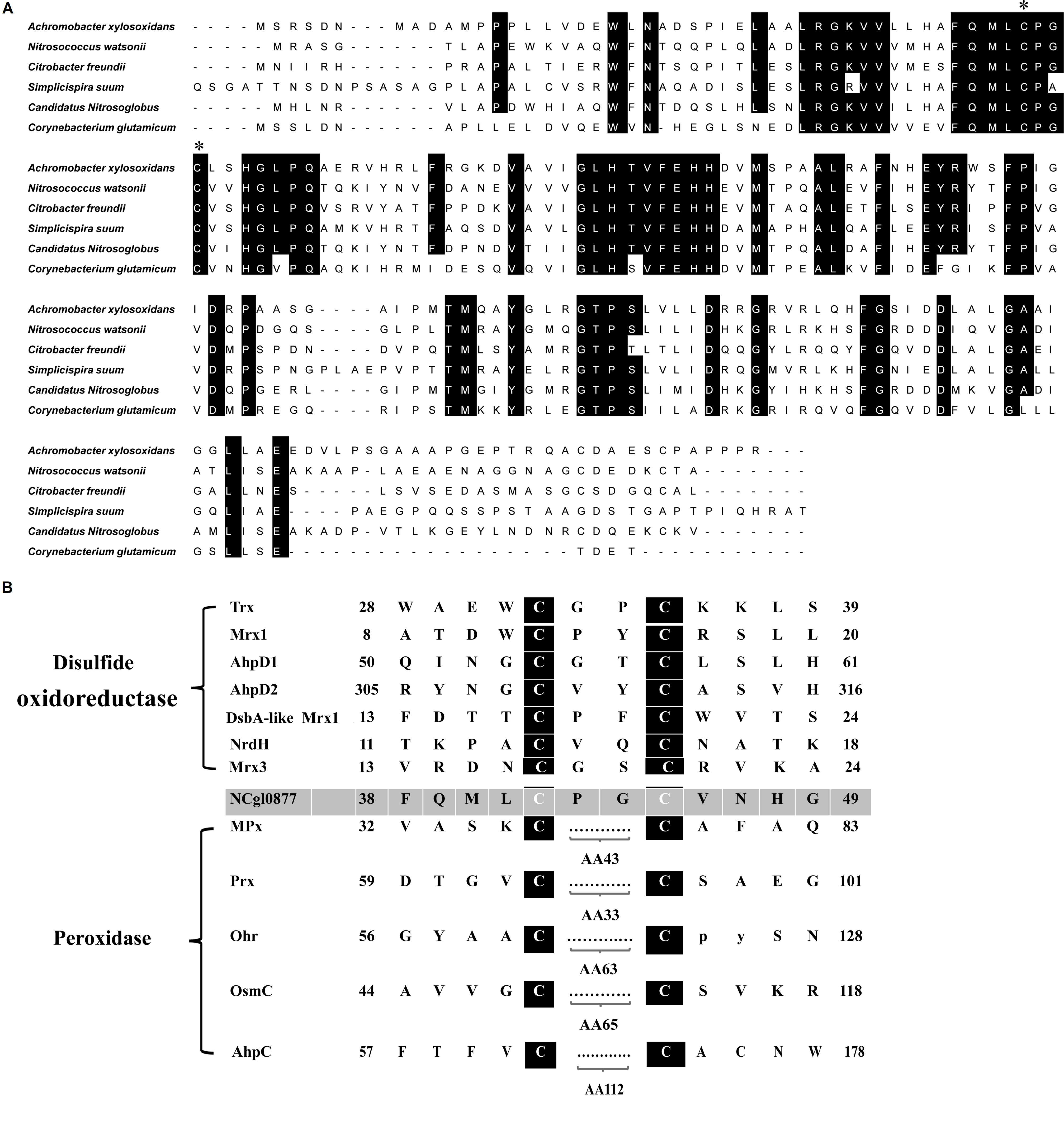


**Fig. S1 Multiple sequence alignment of CgAhp with Ahp in other organisms.** (A) Active site Cys were pointed out by a black star. Residues that were identical in in all or at least 5 of the 6 sequences were depicted on the black background. Reference sequences were retrieved from the NCBI Database, including *Achromobacter xylosoxidans* (ADP19073), *Nitrosococcus watsonii* (ADJ28176); *Citrobacter freundii* (AUV27934); *Simplicispira suum* (AVO42213); *Candidatus nitrosoglobus* (BAW80394); *Corynebacterium glutamicum* (NP_600142). (B) Sequence characterization of disulfide oxidoreductases and peroxidases of *Corynebacterium glutamicum*, and alkyl hydroperoxide reductase (AhpC) of *Mycobacterium tuberculosis*. Sequence alignment between active-site cysteines. Magenta highlights the dicysteine motif. *C. glutamicum* Trx (NP_602283); *C. glutamicum* Mrx1 (NP_600071); *C. glutamicum* AhpD1 (NP_601570); *C. glutamicum* AhpD2 (NP_601633); *C. glutamicum* DsbA-like Mrx1 (NP_601623); *C. glutamicum* NrdH (NP_601732); *C. glutamicum* Mrx3 (NP_599663); *C. glutamicum* NCgl0877 (NP_600142); *C. glutamicum* MPx (NP_601789); *C. glutamicum* Prx (NP_600314); *C. glutamicum* Ohr (NP_599276); *C. glutamicum* OsmC (NP_600290); *M. tuberculosis* AhpC (NP_216944). AA43, AA33, AA63, AA65, and AA112 expressed 43, 33, 63, 65, and 112 amino acids between two cysteines, respectively.

**Reductase activities of CgAhp using different reducing power. A**-**B**. CgAhp activities were measured by recording NADPH oxidation at 340 nm. The reaction mixture contained 50 mM Tris-HCl (pH 8.0), 2 mM EDTA, 250 μM NADPH, 2 μM CgMsrA, 100 mM MetO, and the Trx system (4 μM TrxR and 40 μM Trx) (**A**) or Mrx1 system (500 µM MSH, 4 μM Mtr and 40 μM Mrx1)


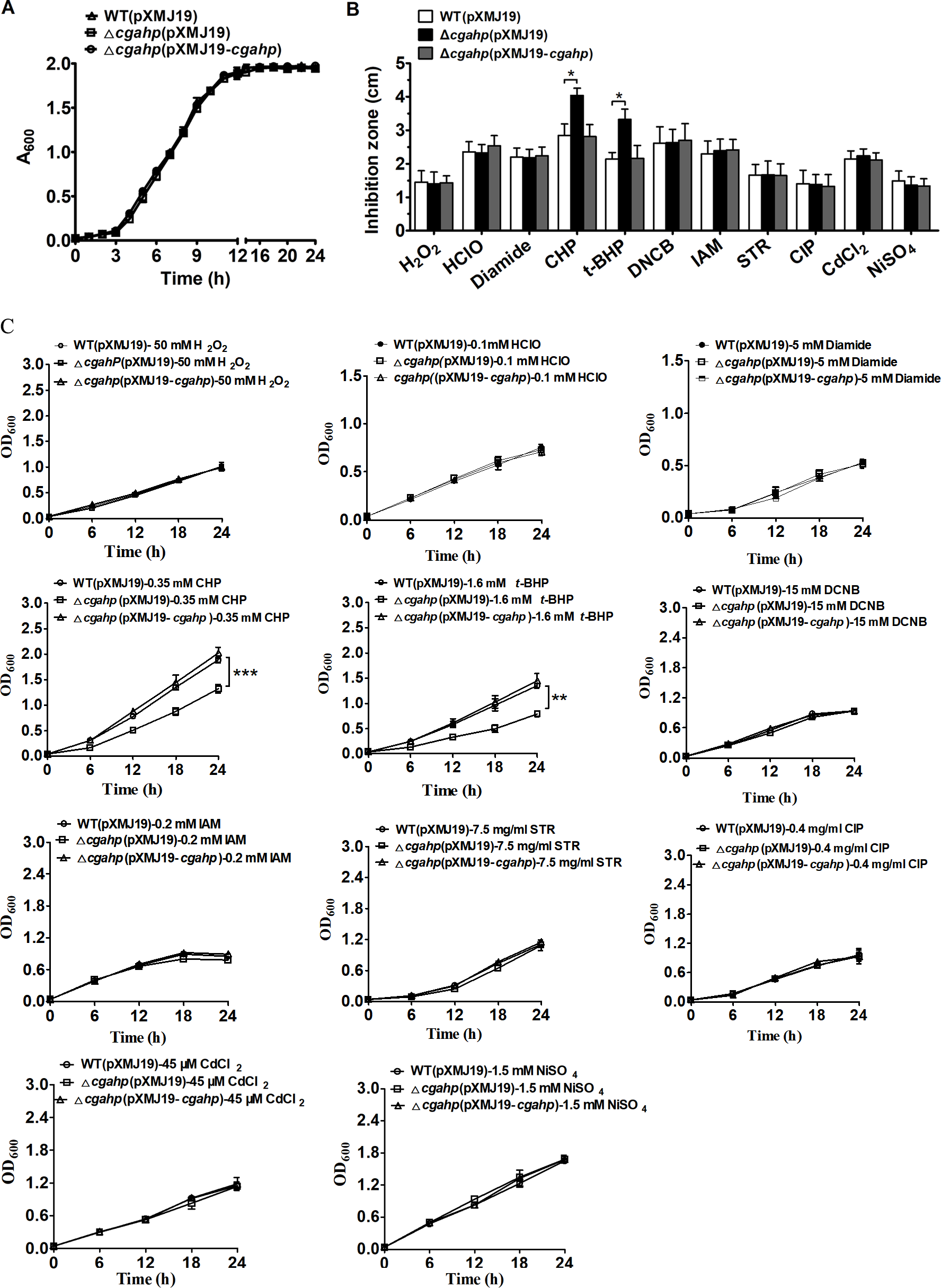


**Fig. S2** **The Δ*cgahp* strains of *C. glutamicum* were more sensitive to** **organic peroxide stress.** (A) Growth curves of the *Corynebacterium glutamicum* RES167 parental strain (WT) containing pXMJ19 [WT(pXMJ19)], Δ*cgahp*(pXMJ19) mutant (the mutant lacking *cgahp* with the empty plasmid pXMJ19), Δ*cgahp*(pXMJ19-*cgahp*) strain (the mutant lacking *cgahp* with the plasmid pXMJ19 containing the wild-type *C. glutamicum* *cgahp* gene) under normal condition. The growth of the indicated strains in LB was monitored by measuring *A*_600_ at indicated time points. (B) The inhibition zone diameter (cm) of WT(pXMJ19), Δ*cgahp*(pXMJ19) mutant and Δ*cgahp*(pXMJ19-*cgahp*) caused by the paper disks (Ø = 5 mm) with oxidative stress-inducing reagents. Data showed the mean and standard error of the 3 samples sets for each reagent. The asterisk indicated a significant correlation between the WT(pXMJ19) and Δ*cgahp*(pXMJ19) mutants at *P ≤ 0.05. (C) Cgahp was required for optimal growth under organic peroxide stress. Growth of indicated strains in LB broth with different stress. Data showed the averages of three independent experiments, and error bars indicated the SDs from three independent experiments. ***, P ≤ 0.001; **, P ≤ 0.01.


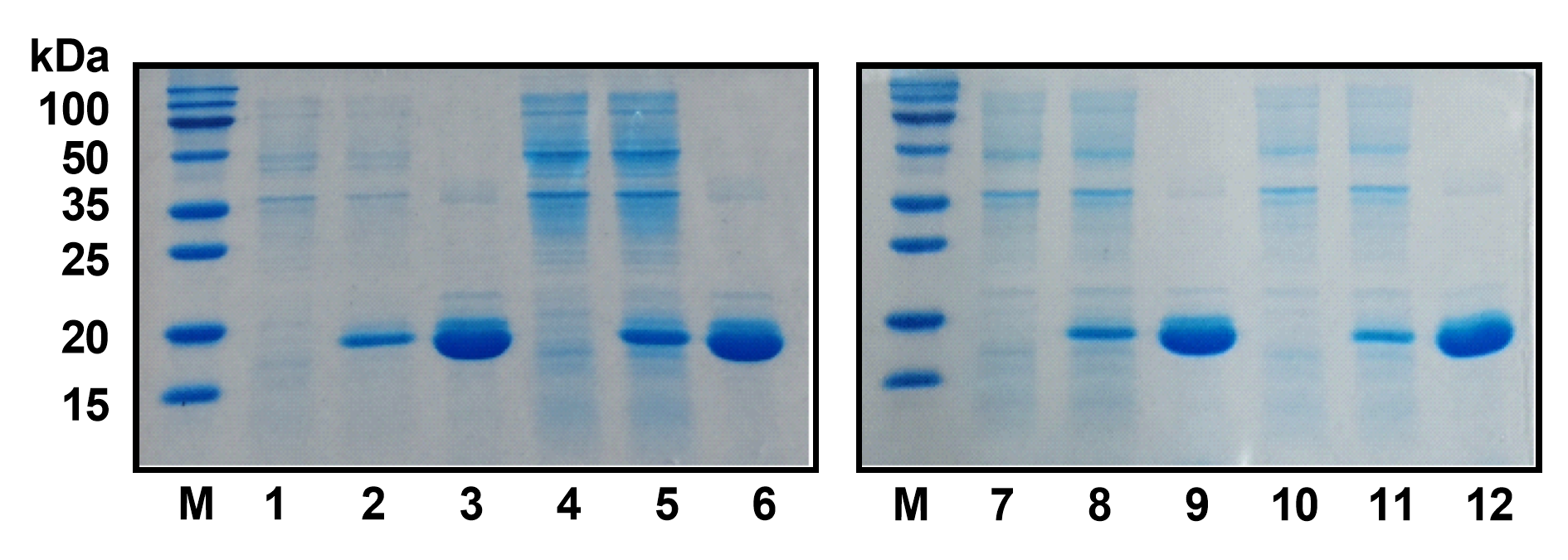


**Fig. S3 SDS-PAGE analysis of purified CgAhp and its variants.**  M, broad-range protein marker; lane 1, crude extract of BL21(pET28a*-cgahp*) strain without IPTG induction; lane 2, crude extract of BL21(pET28a*-cgahp*) strain with induction; lane 3, purified His_6_-CgAhp protein; lane 4, crude extract of BL21(pET28a*-cgahp:C42S*) strain without IPTG induction; lane 5, crude extract of BL21(pET28a*-cgahp:C42S*) strain with induction; lane 5, purified His_6_-CgAhp:C42S protein; lane 7, crude extract of BL21(pET28a*-cgahp:C45S*) strain without IPTG induction; lane 8, crude extract of BL21(pET28a*-cgahp:C45S*) strain with induction; lane 9, purified His_6_-CgAhp:C45S protein; lane 10, crude extract of BL21(pET28a*-cgahp:C42SC45S*) strain without IPTG induction; lane 11, crude extract of BL21(pET28a*-cgahp:C42SC45S*) strain with induction; lane 12, purified His_6_-CgAhp:C42SC45S protein.


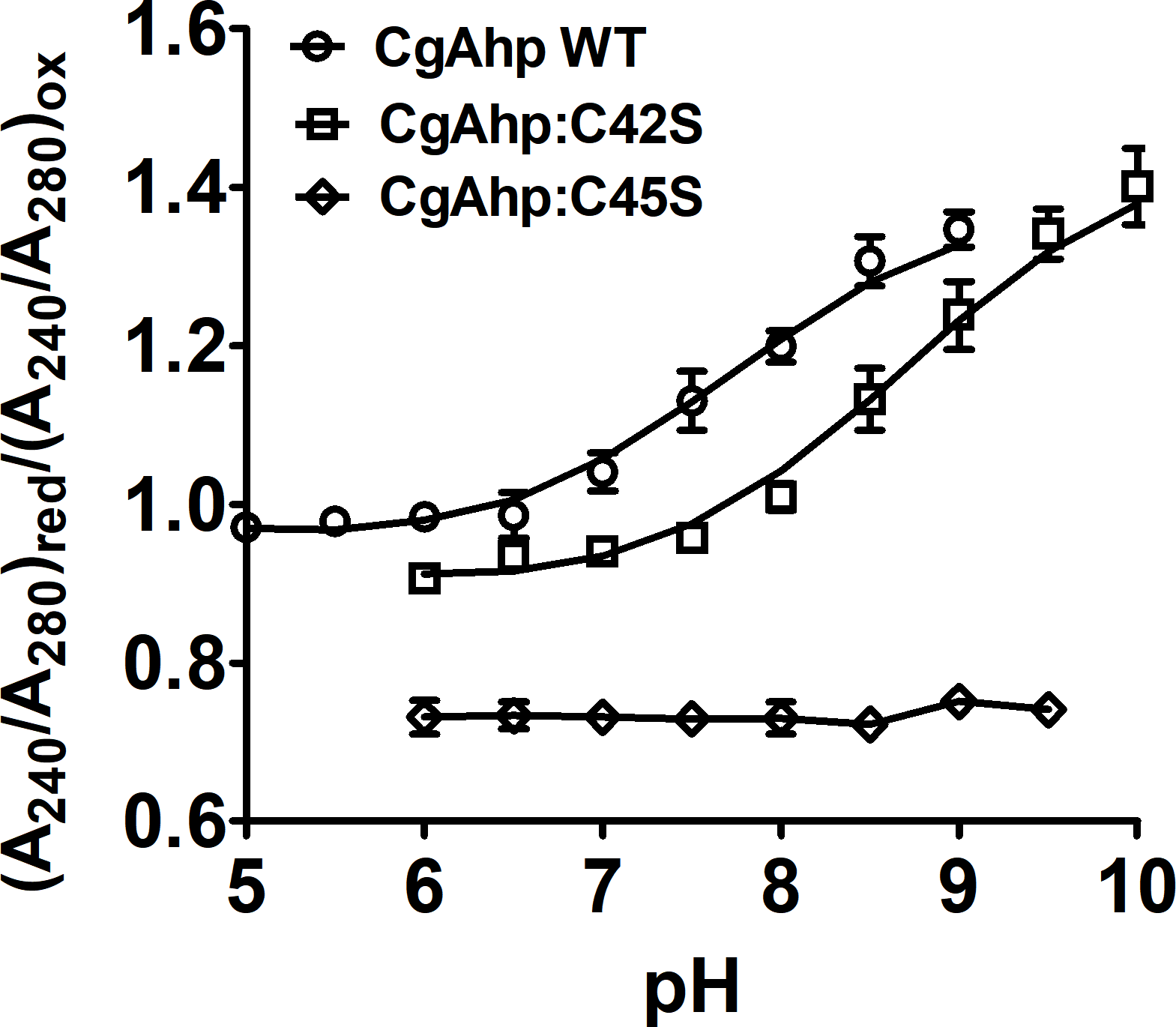


**Fig. S4** **Cys42 was the nucleophilic cysteine of CgAhp**. The p*K*a measurement of the active site cysteines indicates that Cys42 has an unusual p*K*a value lower than 6 and that Cys45 was the resolving cysteine with a p*K*a of 8.39. Ionized thiol groups (R-SH) extinction coefficient at 240 nm was utilized to measure p*K*a values of cysteines of CgAhp WT (○) and the CgAhp:C42S (□) and CgAhp:C45S (◊). The ratio composed by 240/280_red_ and 240/280_ox_ in a pH range of 5.0 to 12 as fitted with the Henderson-Hasselbalch equation.


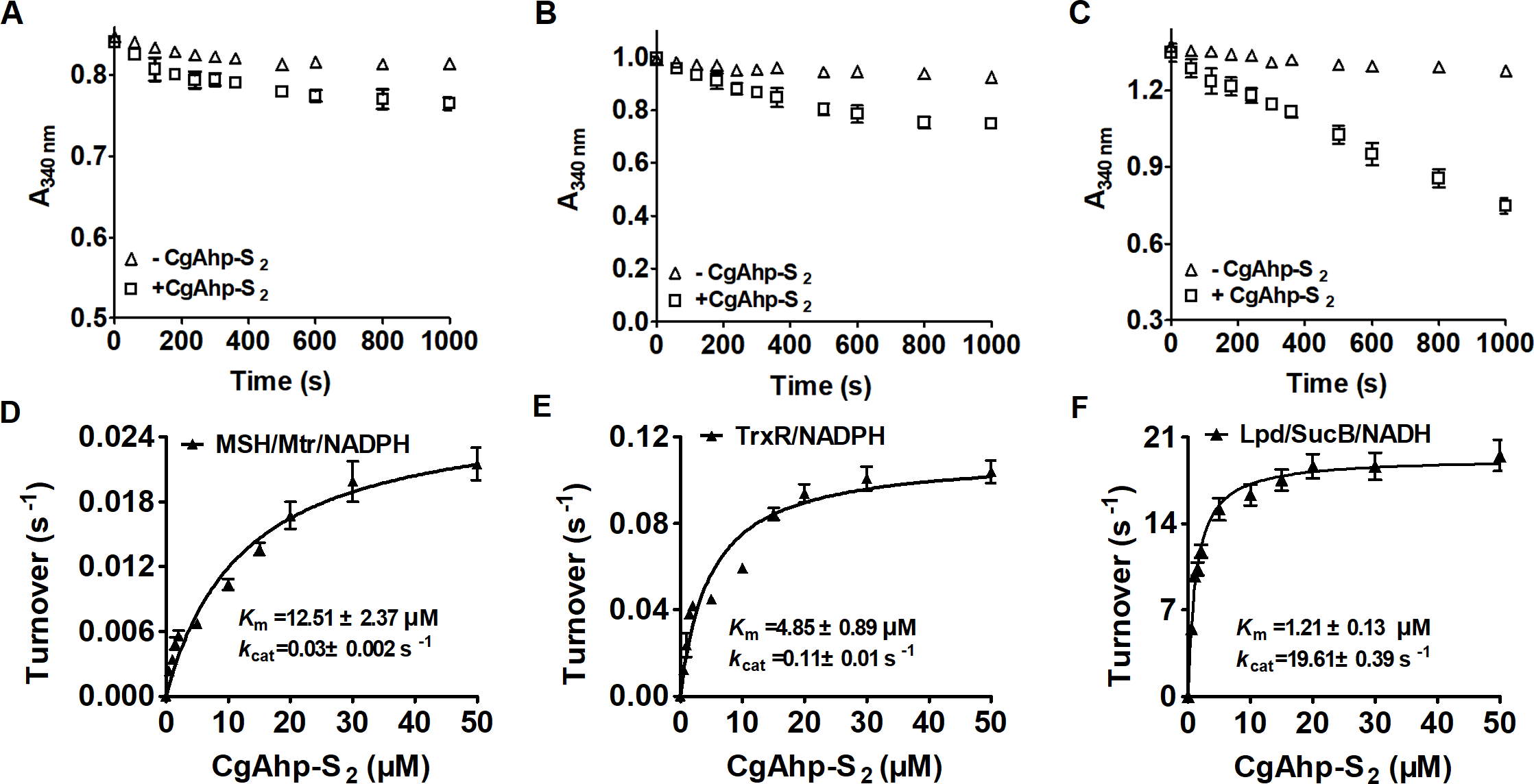


**Fig. S4** **Oxidized CgAhp (CgAhp-S_2_) was preferably reduced by the Lpd/SucB/NADH pathway.** (A-C) CgAhp-S_2_ was added as substrate to the MSH/Mtr/NADPH(A), theTrxR/NADPH (B) and the Lpd/SucB/NADH (C) pathway. A control reaction in the absence of CgAhp-S_2_ was included. The consumption of NADPH or NADH at 340 nm was shown. (D-F) The reduction of CgAhp-S_2_ by the MSH/Mtr/NADPH, the TrxR/NADPH, or Lpd/SucB/NADH pathway was evaluated via Michaelis-Menten steady-state kinetics using the program GraphPad Prism 5. The data were represented as mean ± SD of three independent experiments. Different concentrations of CgDsbA-S_2_ were mixed with a pre-incubated reaction mixture.
